# Supplementary material for: UHRF1 is a mediator of KRAS driven oncogenesis in lung adenocarcinoma
Source: Nat Commun. 2023 Jul 5;14:3966. doi: 10.1038/s41467-023-39591-2 (PMC10322837; doi:10.1038/s41467-023-39591-2)

EXTENDED DATA

UHRF1 is a mediator of KRAS driven oncogenesis in lung adenocarcinoma

Kaja Kostyrko[1,#], Marta Roman[1], Alex G. Lee[1], David R. Simpson[1], Phuong T. Dinh[1], Stanley G. Leung[1], Kieren D. Marini[1], Marcus R. Kelly[2], Joshua Broyde[3], Andrea Califano[3], Peter K. Jackson[2], E. Alejandro Sweet-Cordero[1,#]

1 Division of Pediatric Oncology, Department of Pediatrics, University of California San Francisco. San Francisco, CA, 94153

2 Baxter Laboratory, Department of Microbiology and Immunology, Stanford University School of Medicine, Stanford, California, USA

3 Department of Systems Biology, Irving Cancer Research Center, Columbia University, New York, NY, 10032 USA

# Corresponding authors: Kaja Kostyrko email: kaja.kostyrko@ucsf.edu

and E. Alejandro Sweet-Cordero alejandro.sweet-cordero@ucsf.edu

Supplementary Fig. 1

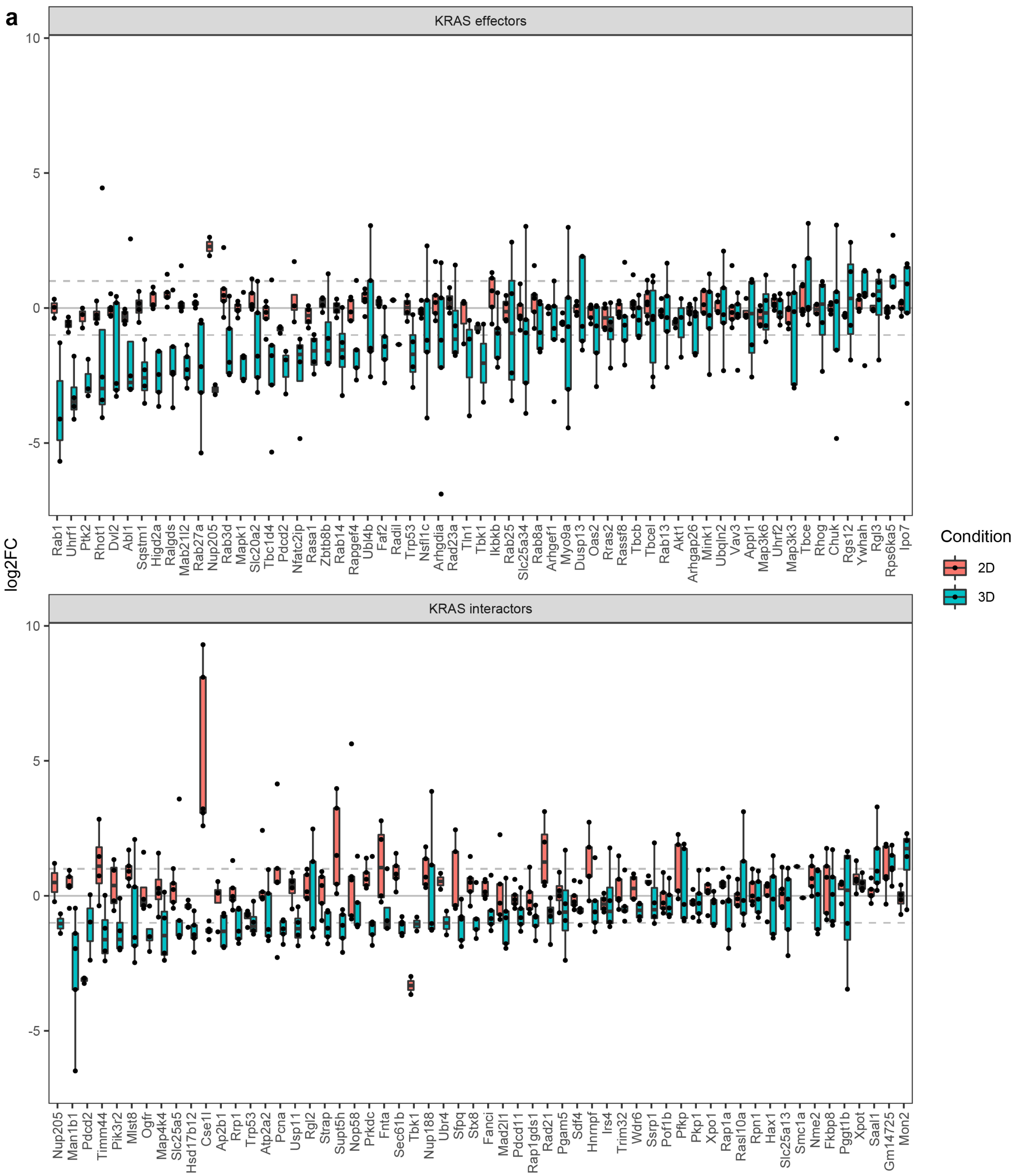

Supplementary Fig. 1

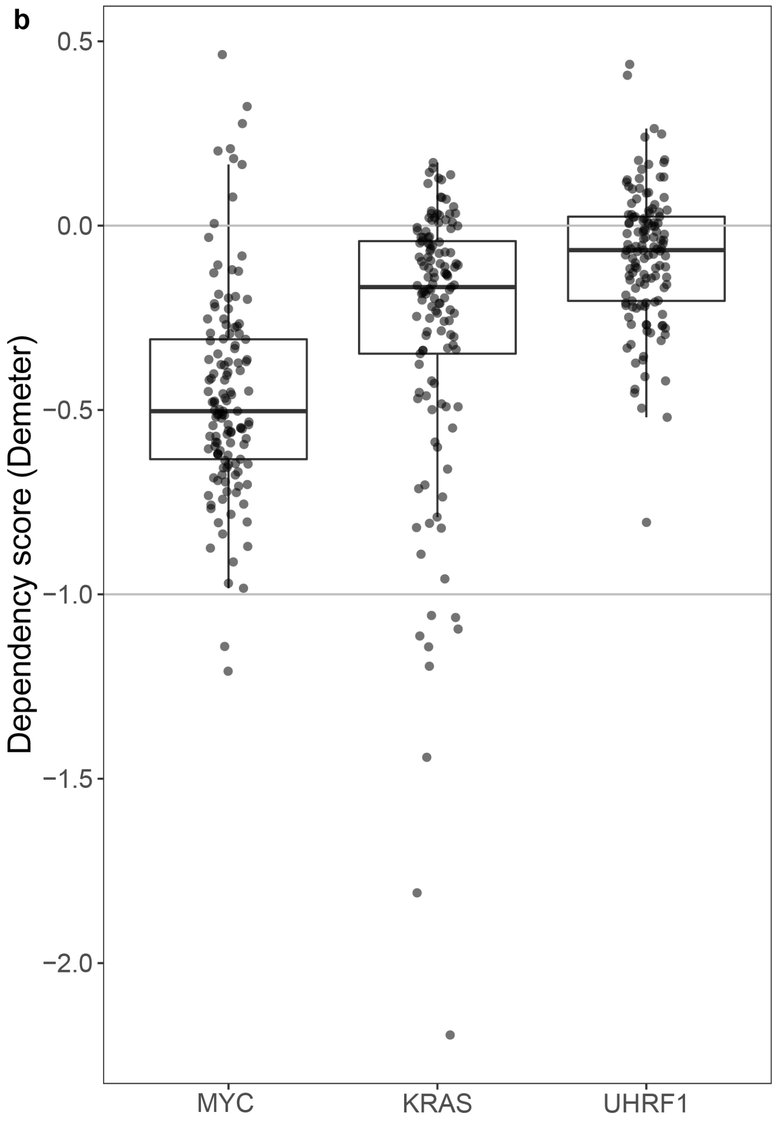

Supplementary Fig. 1. Gene knock-down phenotypes from shRNA screens in primary mouse lung cancer spheroids and adherent LKR10 cells. a, Plots show log2FC values in 3D (blue) screens in primary spheroids and 2D (red) screens in LKR10 cells. Top - shRNA library targeting KRAS effectors, bottom- shRNA library targeting KRAS interactors. Each point represents one hairpin. **b**, Dependency scores (estimated using the Demeter2 model) for UHRF1, KRAS, and MYC across all lung cancer cell lines. RNAi screen data[1,2] downloaded from https://depmap.org/portal/. For boxplots lower whisker is equal to smallest observation greater than or equal to lower hinge - 1.5 * IQR, lower hinge is 25% quantile, 50% quantile is the median value, upper hinge is 75% quantile, upper whisker is equal to the largest observation less than or equal to upper hinge + 1.5 * IQR.

1. Tsherniak, A. *et al.* Defining a Cancer Dependency Map. *Cell* **170**, 564-576.e16 (2017).

2. McDonald, E. R. *et al.* Project DRIVE: A Compendium of Cancer Dependencies and Synthetic Lethal Relationships Uncovered by Large-Scale, Deep RNAi Screening. *Cell* **170**, 577-592.e10 (2017).

Supplementary Fig. 2

a

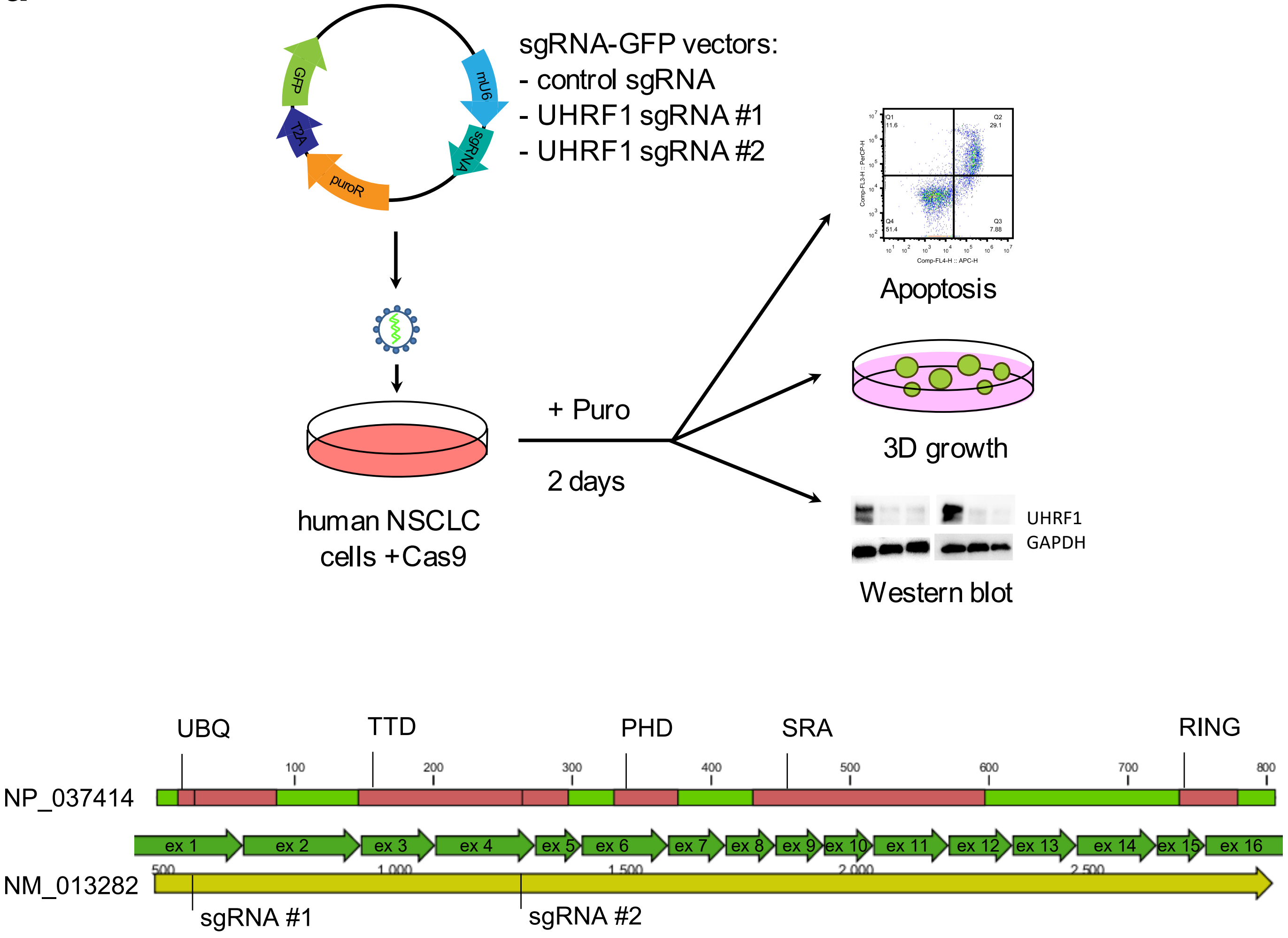

b

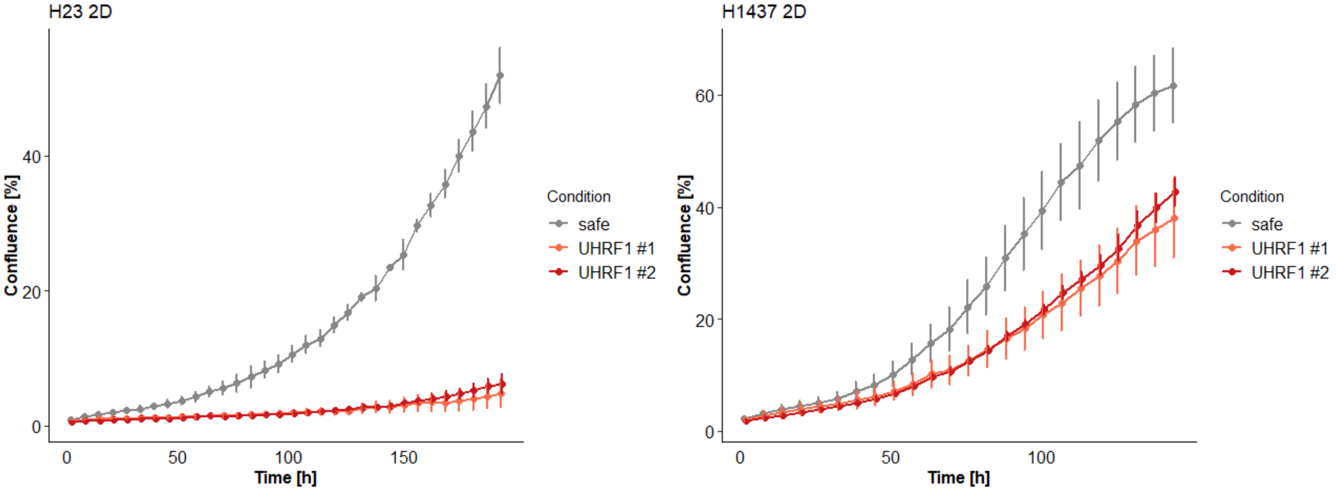

Supplementary Fig. 2

c

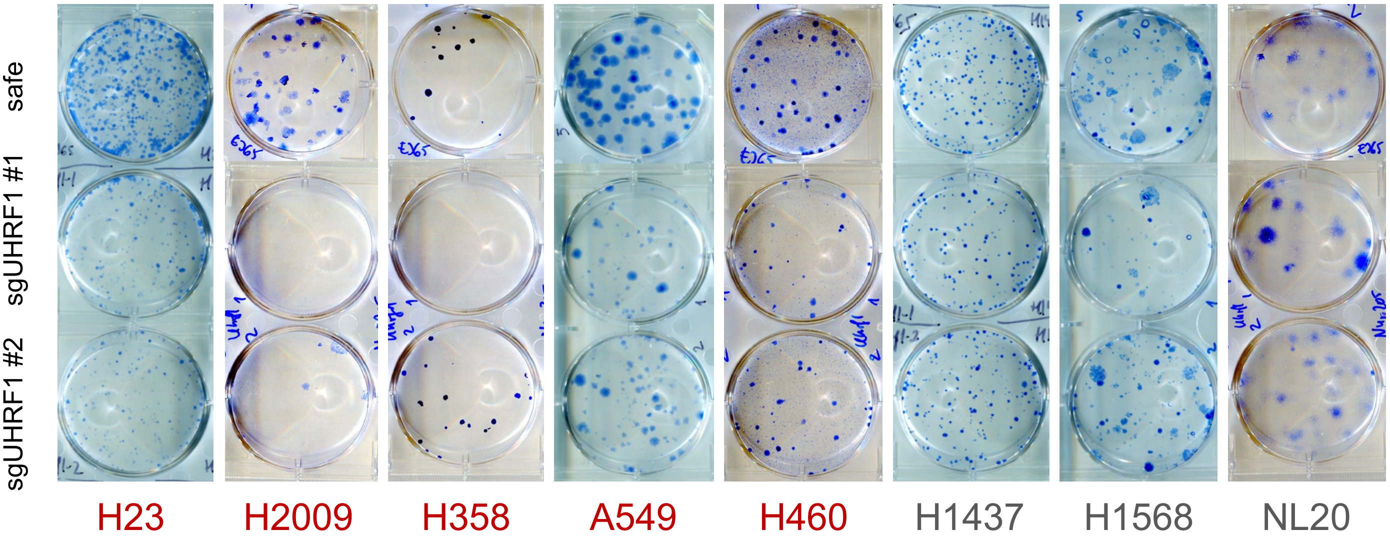

d

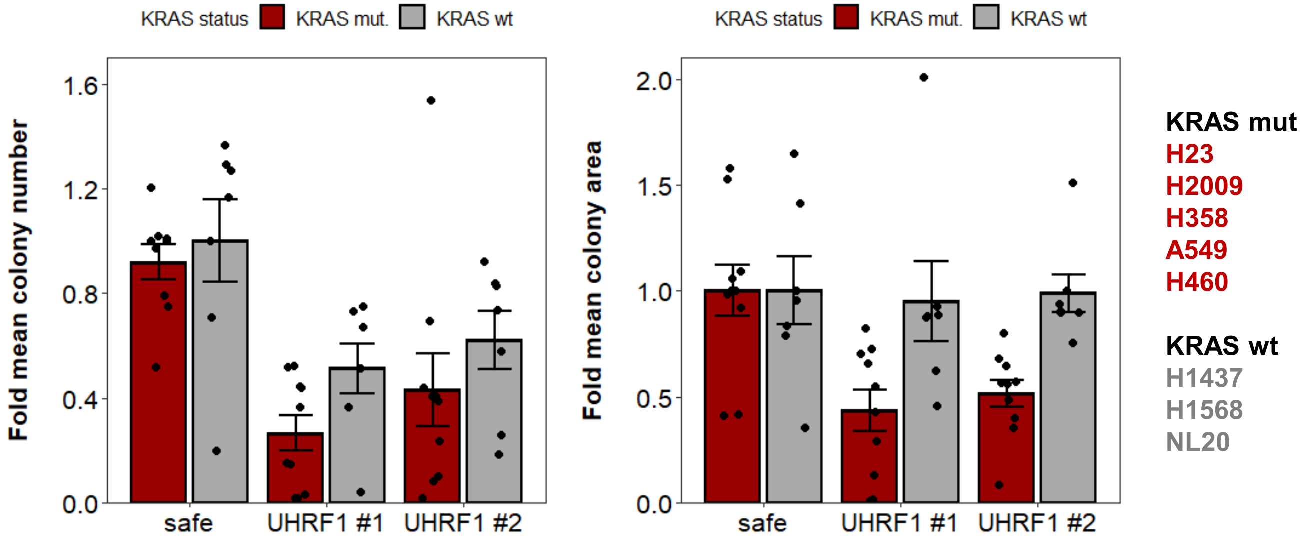

Supplementary Fig. 2

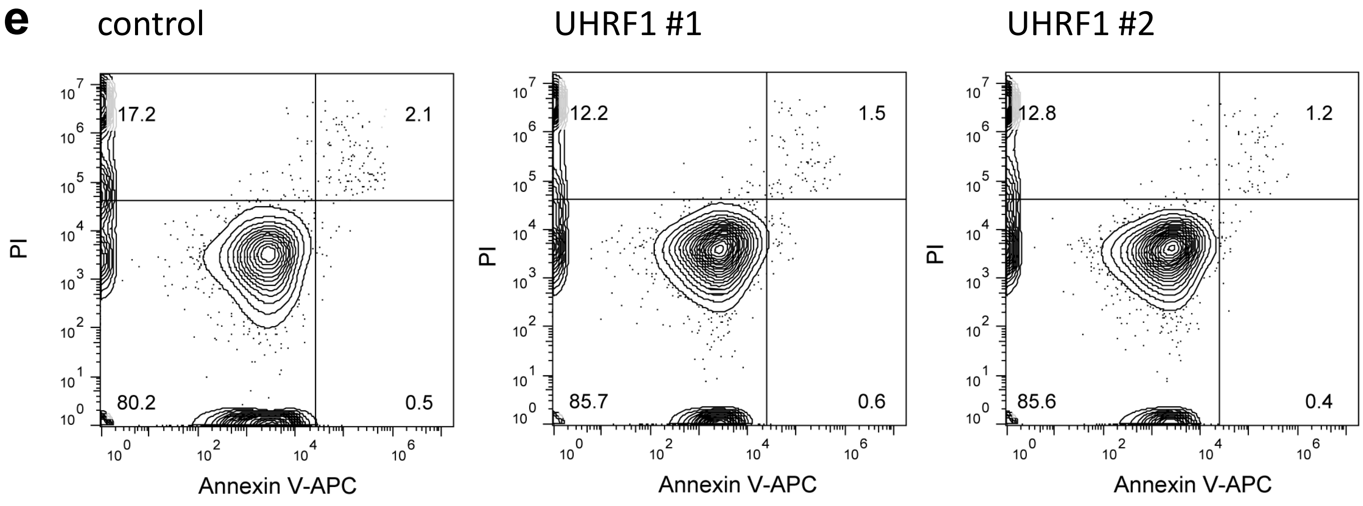

Gating strategy for flow cytometry

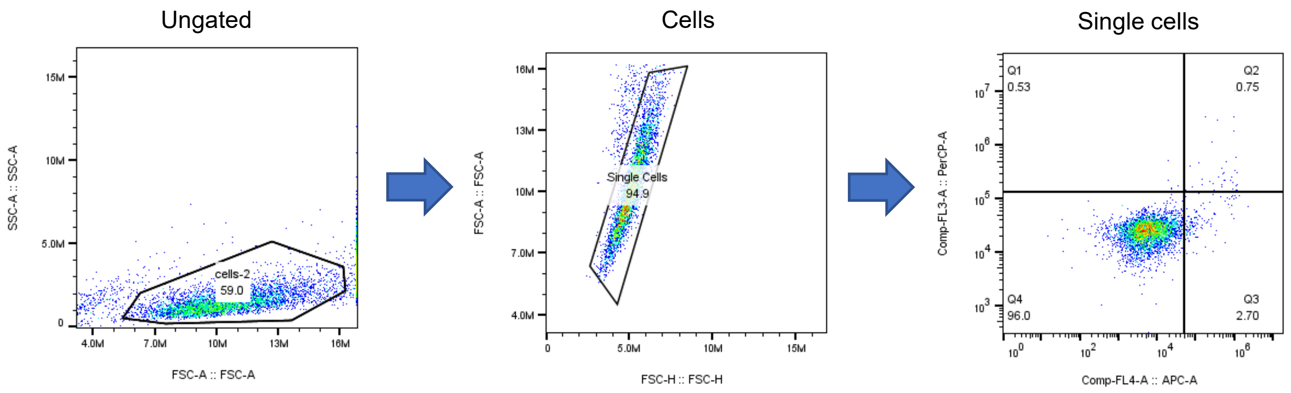

Supplementary Fig. 2

f

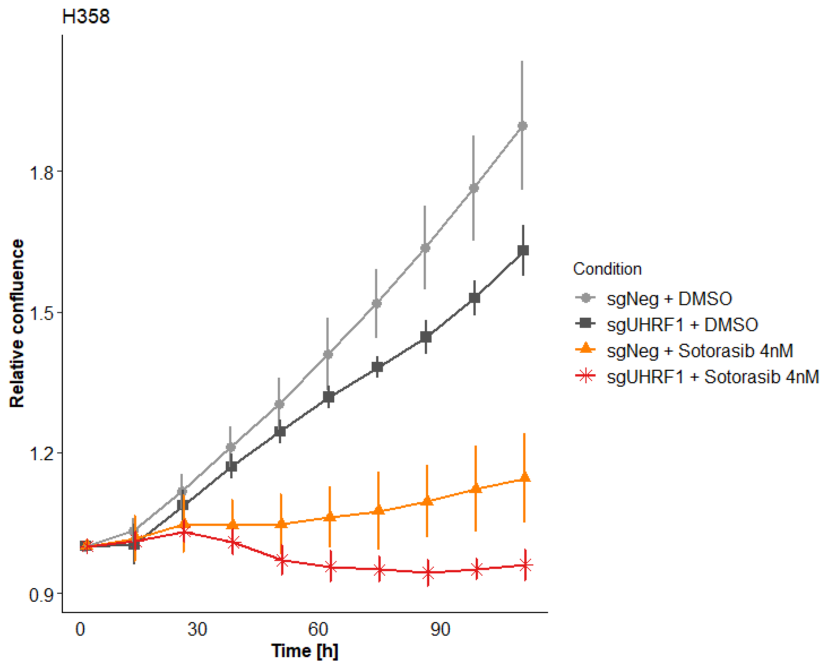

Supplementary Fig. 2. Effect of UHRF1 loss on 2D growth of KRAS mutant and KRAS wild type human cell lines. a, Top - Schematic of in vitro CRISPR/Cas9 experiments in human lung cancer cell lines. Bottom - The sequence of human UHRF1. Protein sequence is indicated in light green with functional domains in red. DNA coding sequence is indicated in yellow, coding exons are represented as dark green arrows. The target sites of the two sgRNAs used in this study have been indicated on the DNA coding sequence. **b**, 2D proliferation curves from a representative experiment in two Cas9-expressing lung cancer cell lines infected with sgRNAs against UHRF1 (UHRF1 #1, UHRF1 #2) or a control ("safe") sgRNA. H23 cells (KRAS G12C, left) and H1437 cells (KRAS wt, right). Proliferation assays were performed using the Incucyte with n=3 technical replicates. Curves represent means with standard deviation error bars. The experiment has been repeated 3 times. **c**, Representative mages of 2D colony formation assays across 8 Cas9-expressing cell lines infected with the indicated sgRNAs; 5 KRAS mutant NSCLC cell lines (H23, H2009, H358, A549, H460), 2 KRAS wild type NSCLC cell lines (H1437, H1568), and 1 HBEC line (NL20). **d**, Quantification of colony number (left) and area (right) in 2D colony formation assays; n=7 biological replicates for KRAS wild type cell lines and n=10 for KRAS mutant cell lines. Bars represent mean values with one standard deviation error bars. **e**, Top - Flow cytometry of a HBEC line (NL20 cells) expressing Cas9 and transduced with lentiviral vectors expressing the indicated sgRNAs. Cells were co-stained with Annexin V-APC and propidium iodide (PI). Bottom – Gating strategy for flow cytometry plots. **f**, 3D growth curves from a representative experiment in H358 cells expressing Cas9 and the indicated sgRNAs treated with either DMSO or 4nM of Sotorasib. Proliferation assays were performed using the Incucyte with n=3 technical replicates. Curves represent means with standard deviation error bars. The experiment has been repeated 3 times. Source data are provided as a Source data file.

Supplementary Fig. 3

a

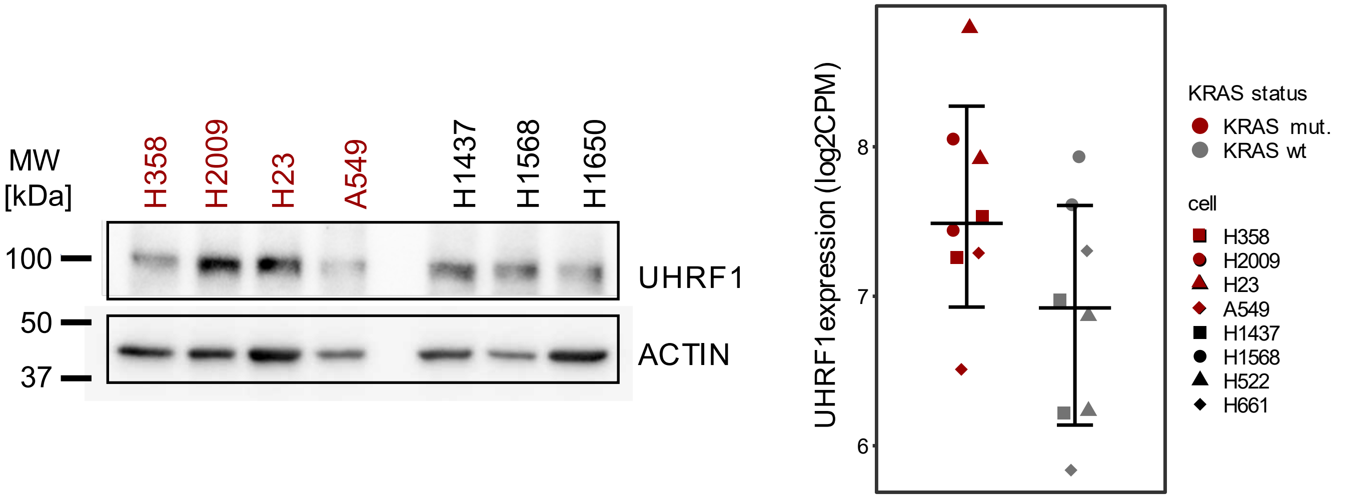

b

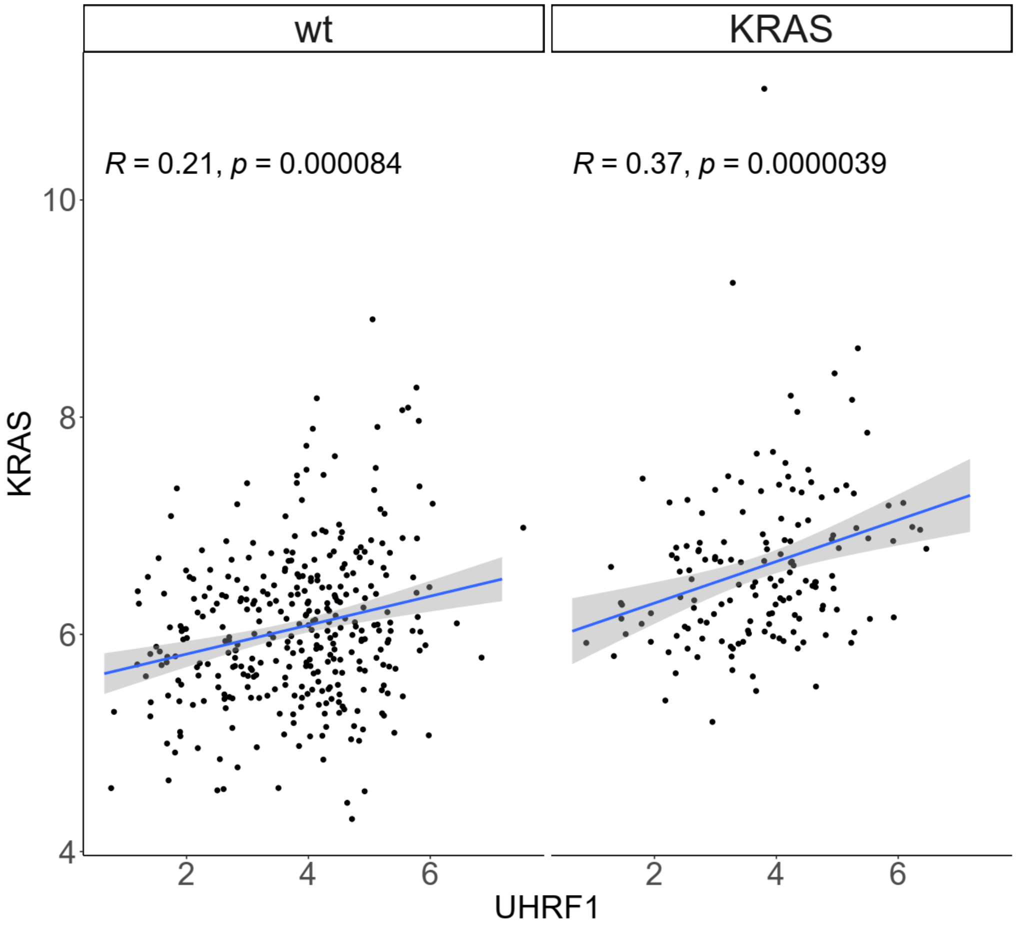

Supplementary Fig. 3

c

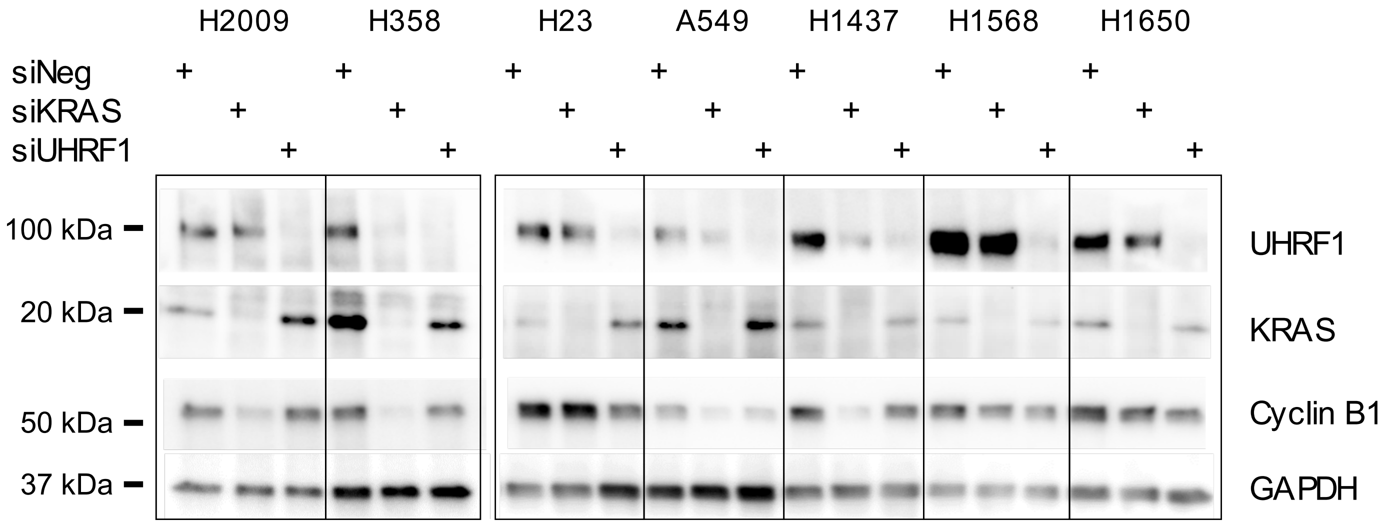

d

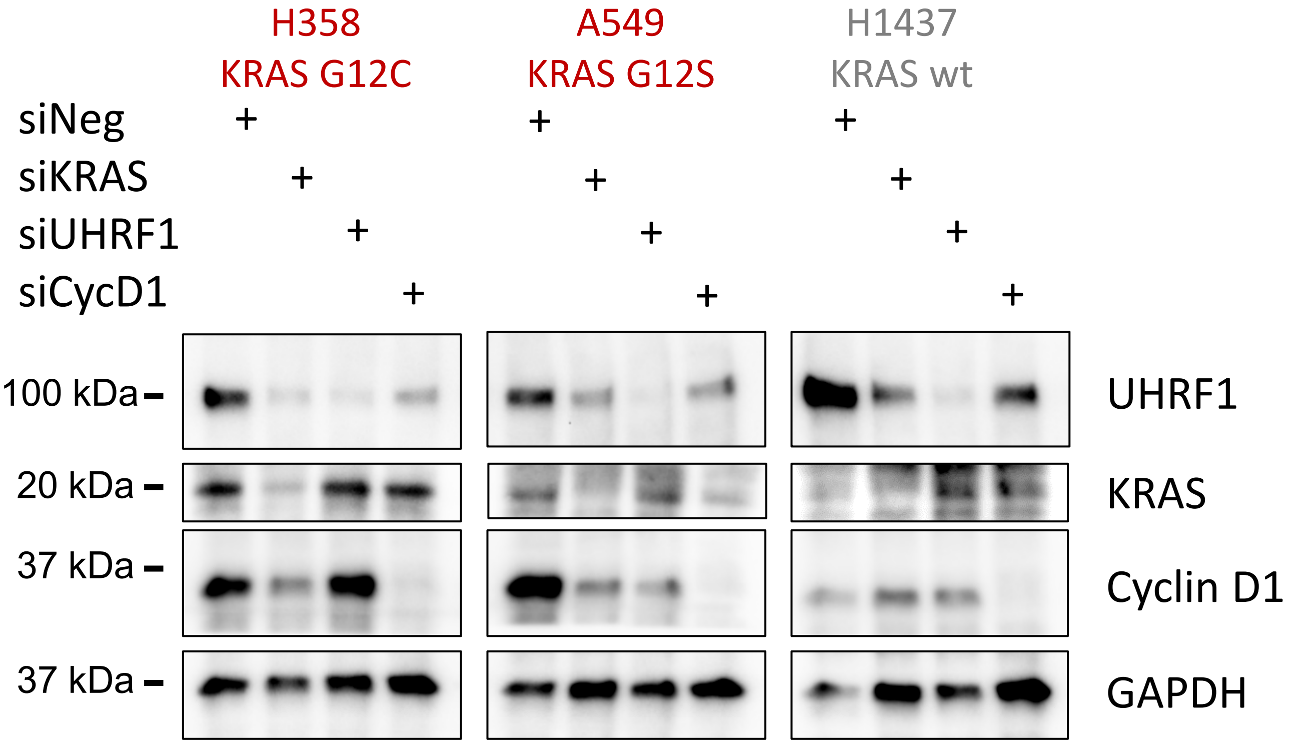

Supplementary Fig. 3

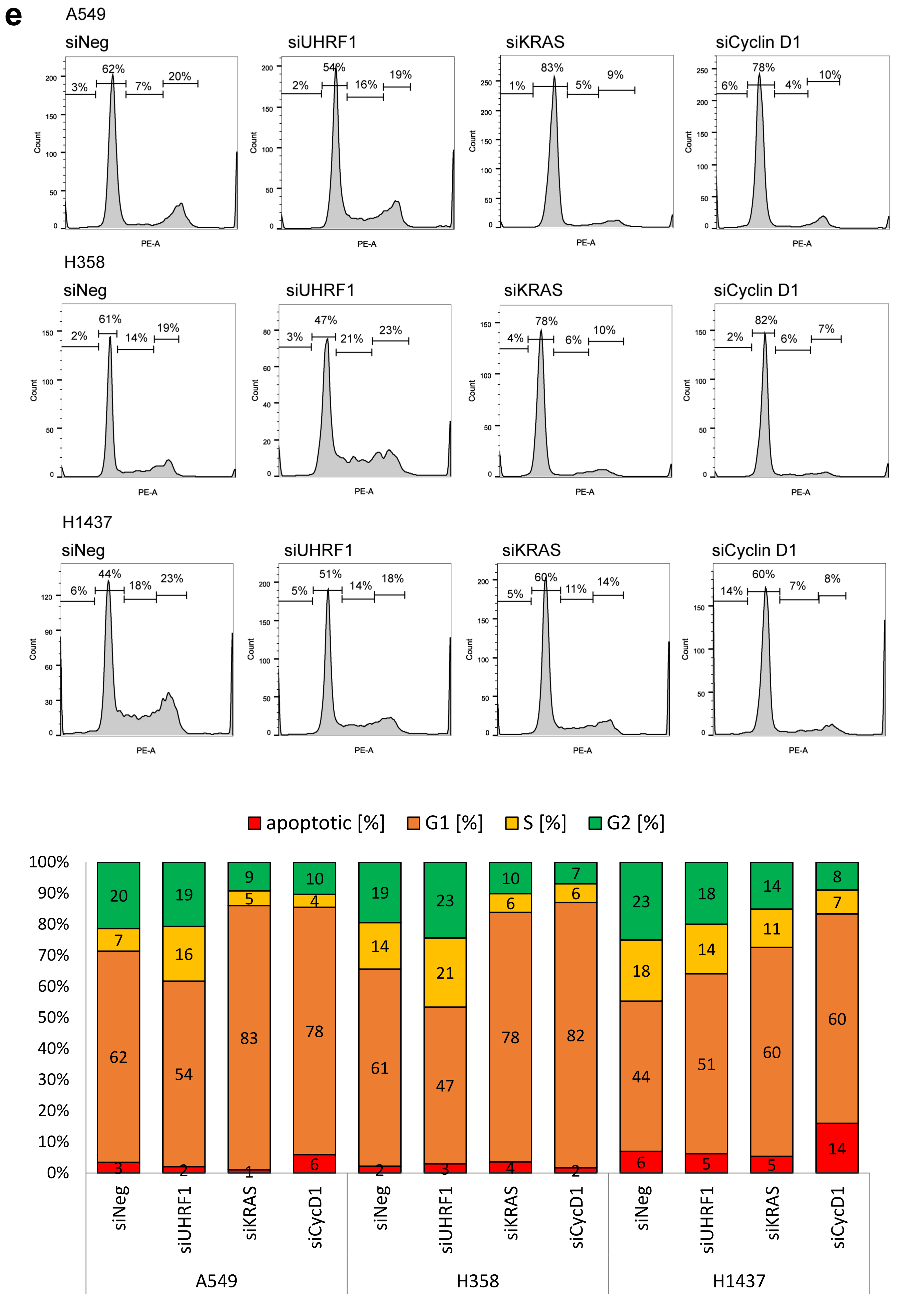

Supplementary Fig. 3

f

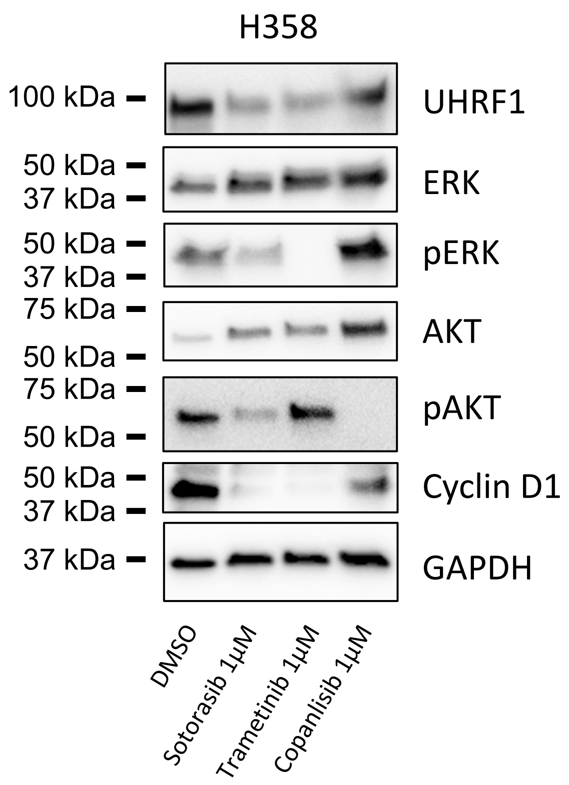

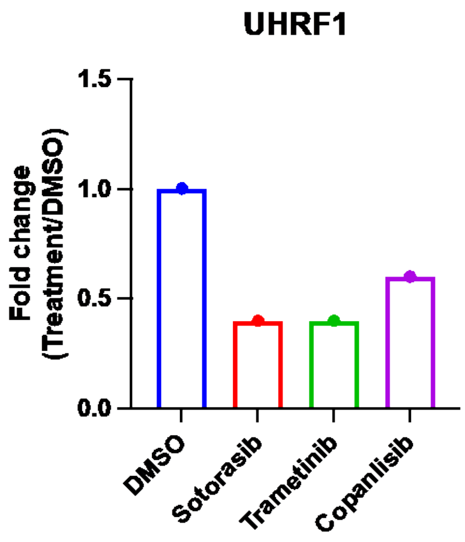

Supplementary Fig. 3

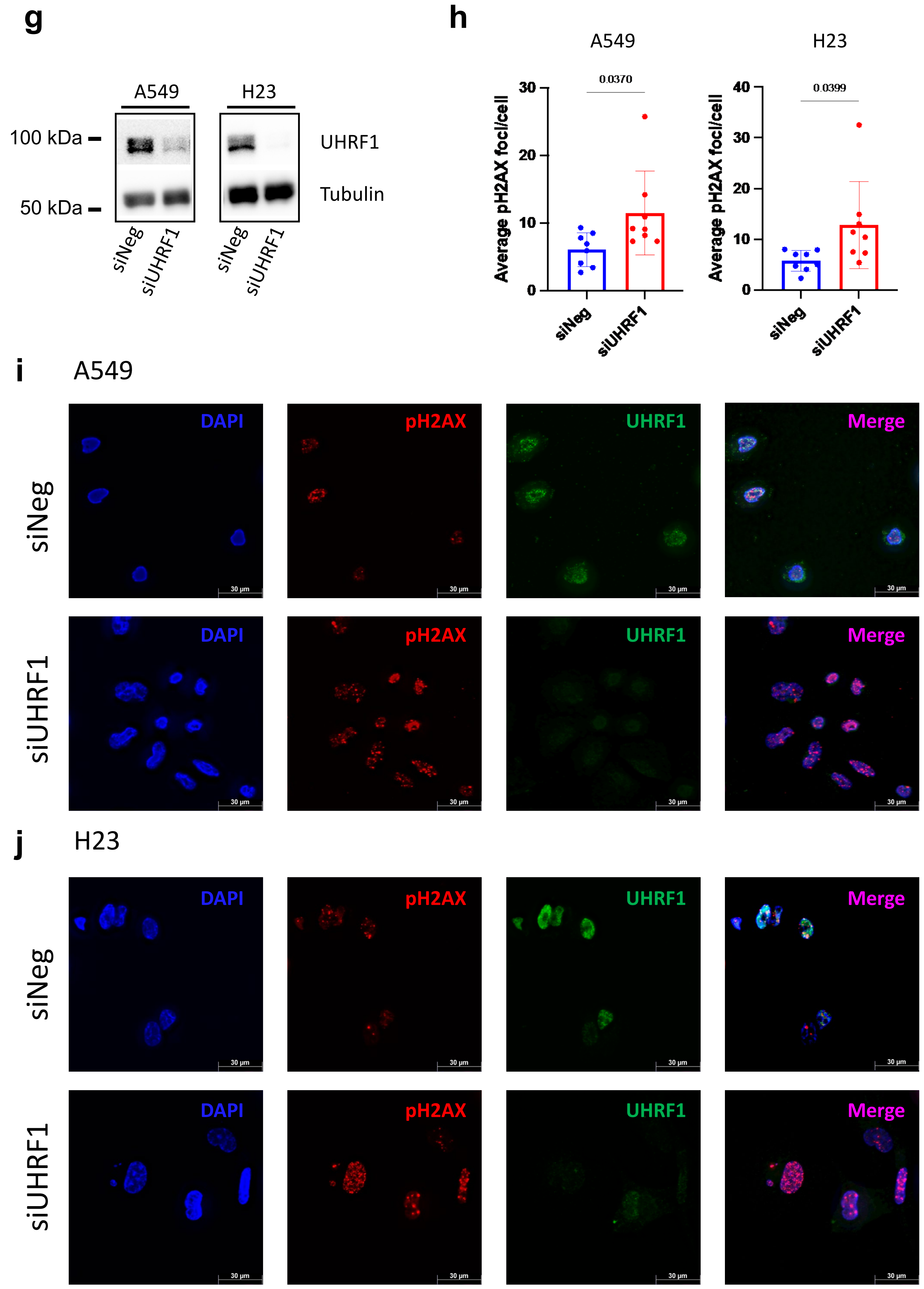

Supplementary Fig. 3

Supplementary Fig. 3. UHRF1 depletion leads to accumulation of cells in the S phase of the cell cycle. a, Left - western blot of UHRF1 protein expression in KRAS mutant (H358, H2009, H23, A549) and KRAS wt NSCLC cell lines (H1437, H1568, H1650). Right - UHRF1 RNA expression in 4 KRAS mutant cell lines (H358, H2009, H23, A549) and 4 KRAS wt cell lines (H1437, H1568, H522, H661); n=8 biological replicates per group; p-value = 0.058 by two sided unpaired Student's t-test between KRAS mutant and KRAS wt cell lines. **b**, Correlation of UHRF1 and KRAS expression (log2CPM) in KRAS mutant and KRAS wild type subsets of the LUAD dataset (TCGA). Linear trend lines were generated using a linear model, shaded confidence regions represent CI=0.95. **c**, Representative western blot image showign protein levels of UHRF1, KRAS, Cyclin B1 and GAPDH in 7 lung cancer cell lines (4 KRAS mut – H2009, H358, H23, A549 and 3 KRAS wt – H1437, H1568, H1650) transfected with the indicated siRNAs. Experiment has been repeated 4 times. **d**, Representative western blot image showing protein levels of UHRF1, KRAS, Cyclin D1 and GAPDH in 3 lung cancer cell lines (2 KRAS mut – H358, A549 and 1 KRAS wt – H1437) transfected with the indicated siRNAs. Protein extracts were collected 72h post siRNA transfection. Replicate wells were collected for cell cycle analysis shown in panel e. Experiment has been repeated 3 times. **e**, Cell cycle analysis of matched samples from panel d. Cells were collected 72h post siRNA transfection, fixed and stained with propidium iodide and analyzed by flow cytometry. Bottom – quantification of cell subpopulations in flow cytometry analysis. **f**. Left - Representative western blot image showing the level of UHRF1, pERK/ERK, pAKT/AKT, Cyclin D1 and GAPDH in extracts from H358 cells treated with the indicated inhibitors for 24h. Right – quantification of the UHRF1 signal using ImageJ from that experiment. The experiment was performed two times. **g**, Western blot confirming the absence of UHRF1 protein in cells treated with UHRF1 siRNAs. Matched samples were used for IF shown in panels g-j. **h**, Quantification of pH2AX foci in A549 and H23 cells transfected with UHRF1 siRNA in n=8 individual images per condition. Bars represent means with one standard deviation error bars. **i**, IF images of A549 cells transfected with control (siNeg) or UHRF1 siRNA (siUHRF1). Images were acquired with a 100x objective. **j**, IF images of H358 cells transfected with control (siNeg) or UHRF1 siRNA (siUHRF1). Images were acquired with a 100x objective. Source data are provided as a Source data file.

Supplementary Fig. 4

a

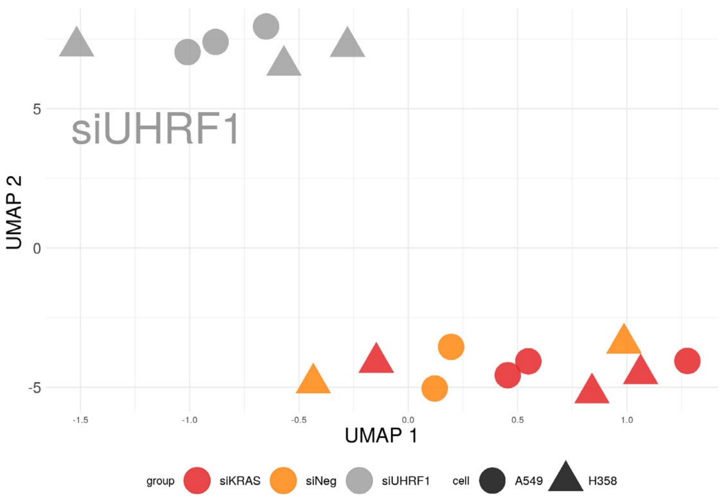

b

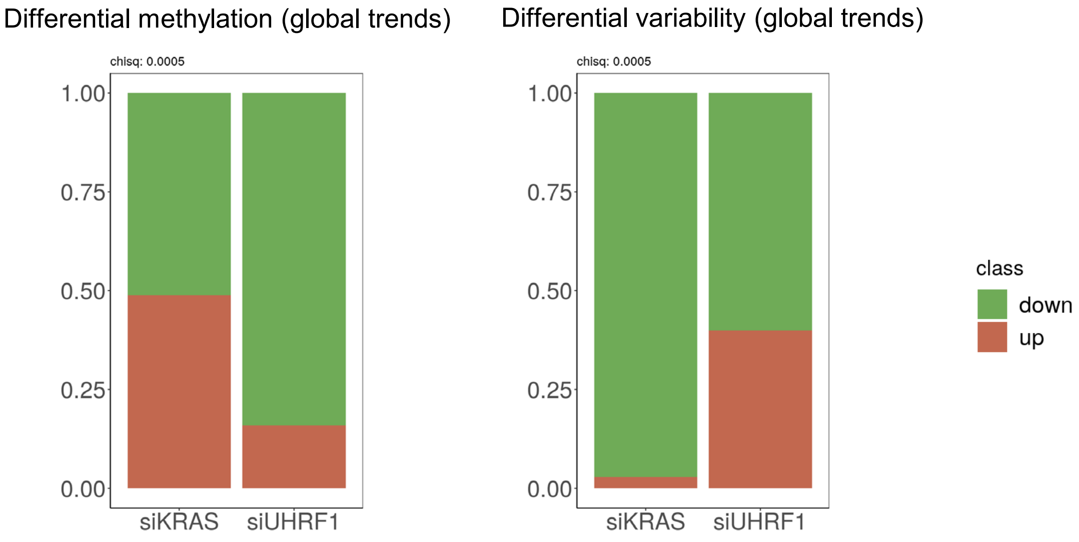

c

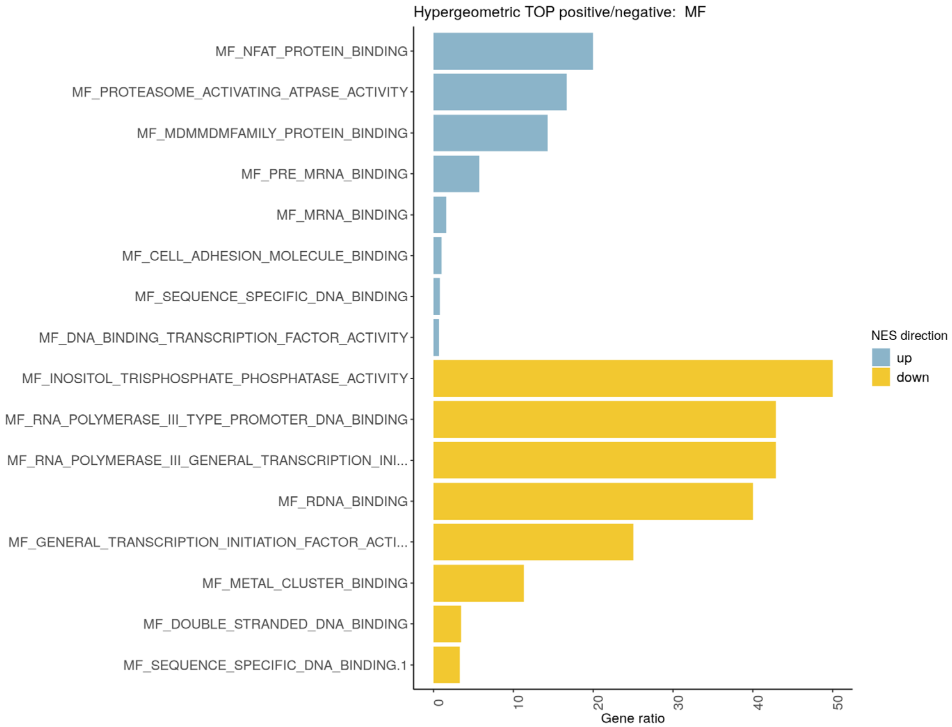

Supplementary Fig. 4

d

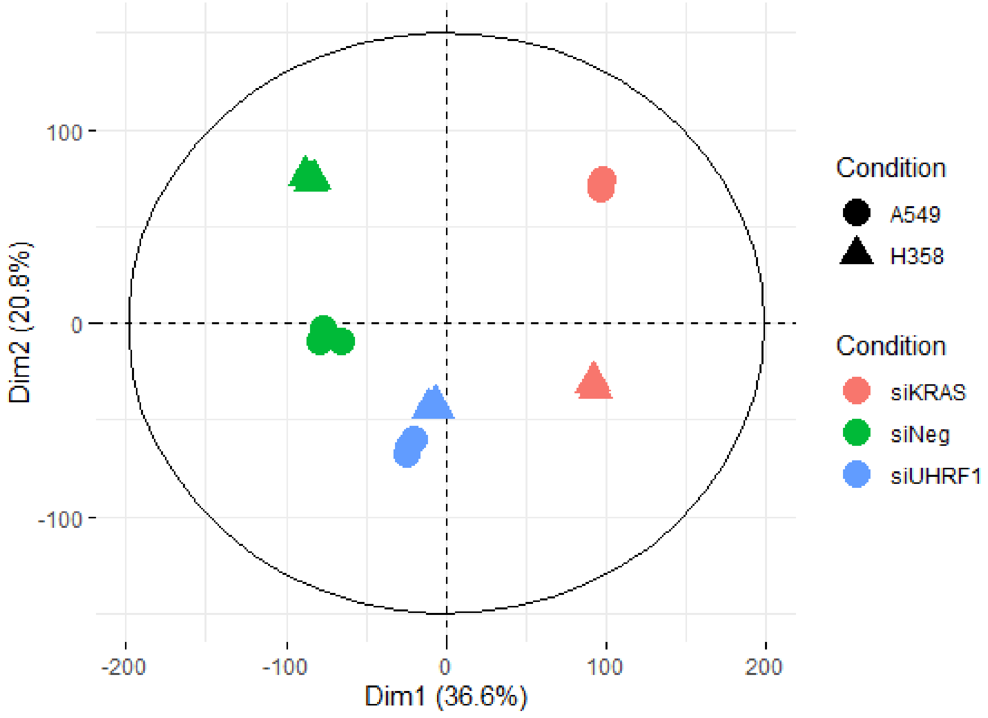

e

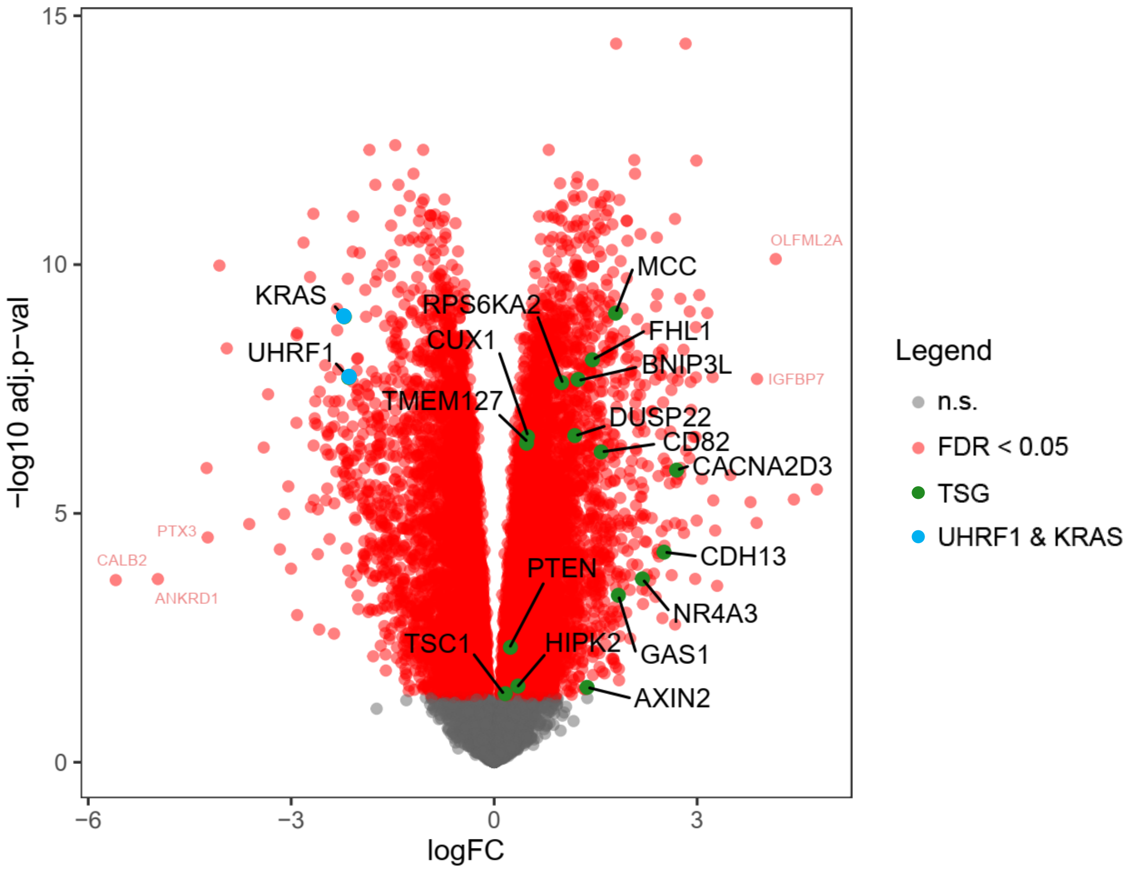

Supplementary Fig. 4

f

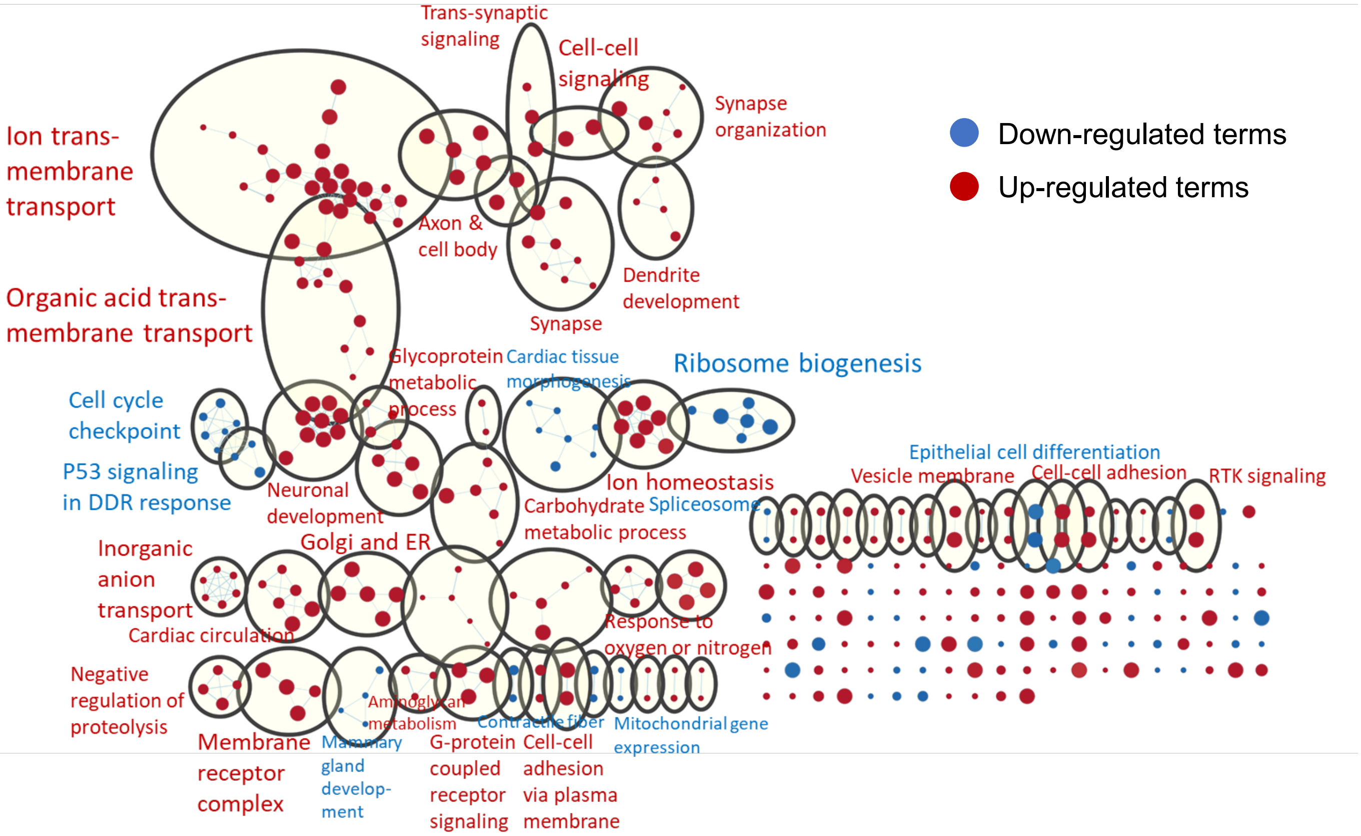

g

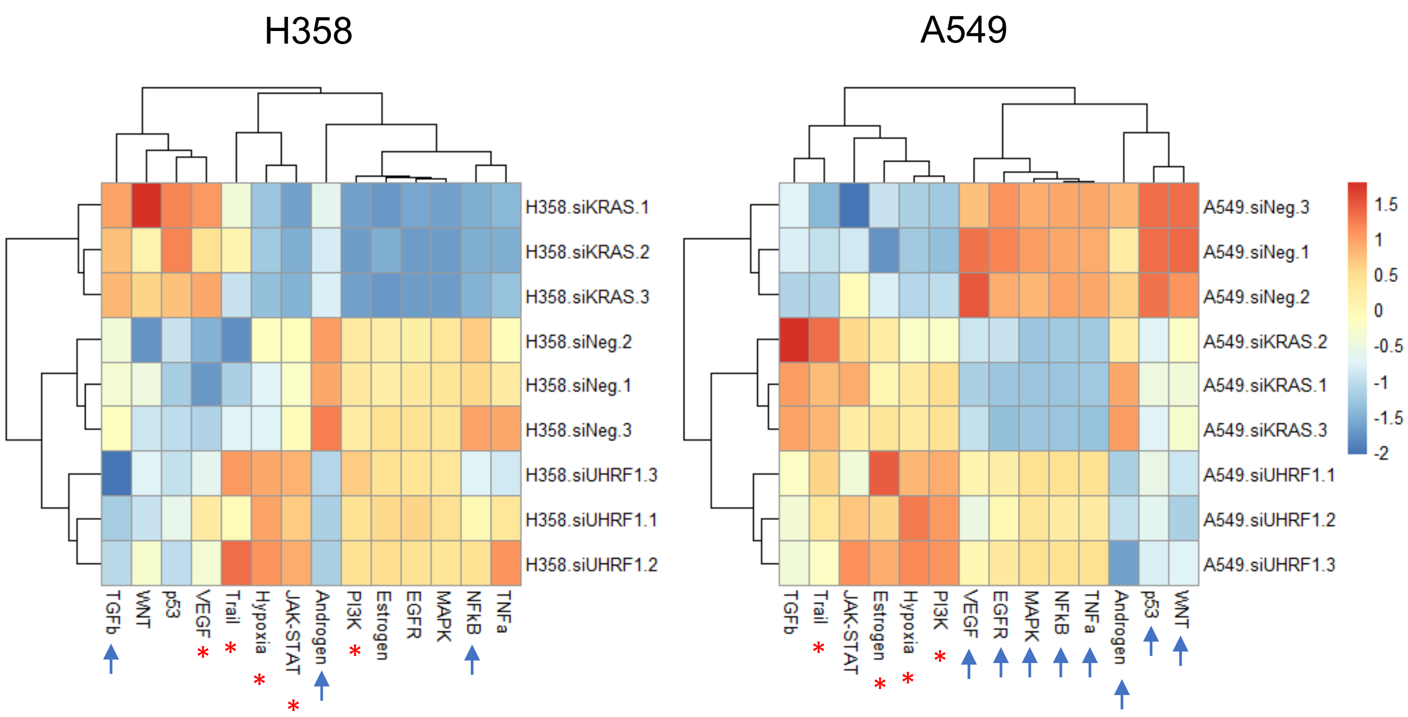

Supplementary Fig. 4

h

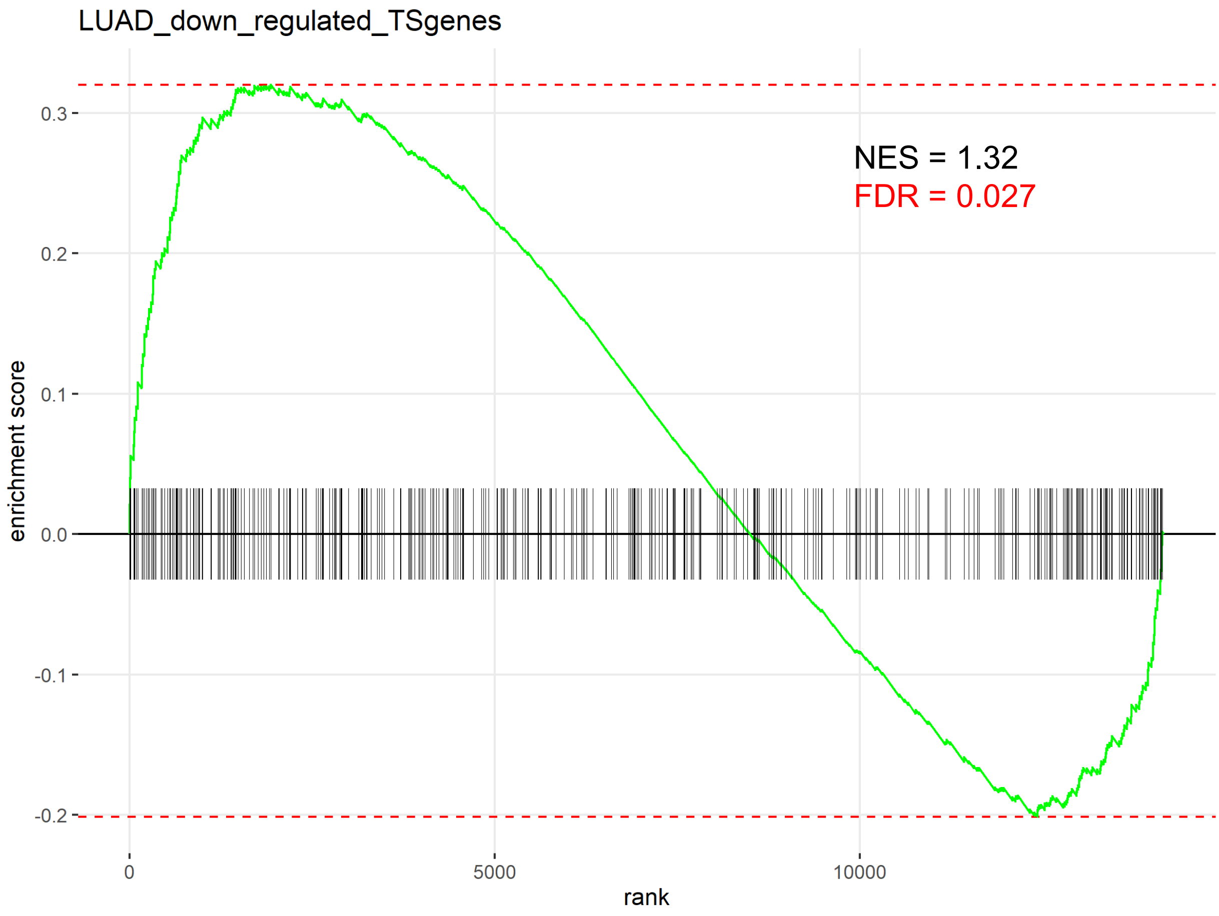

i

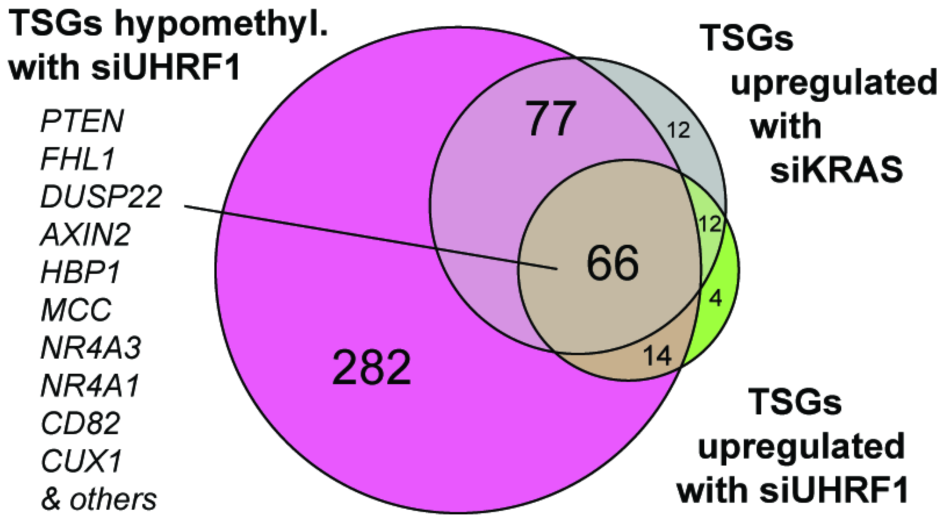

Supplementary Fig. 4

j HIPK2

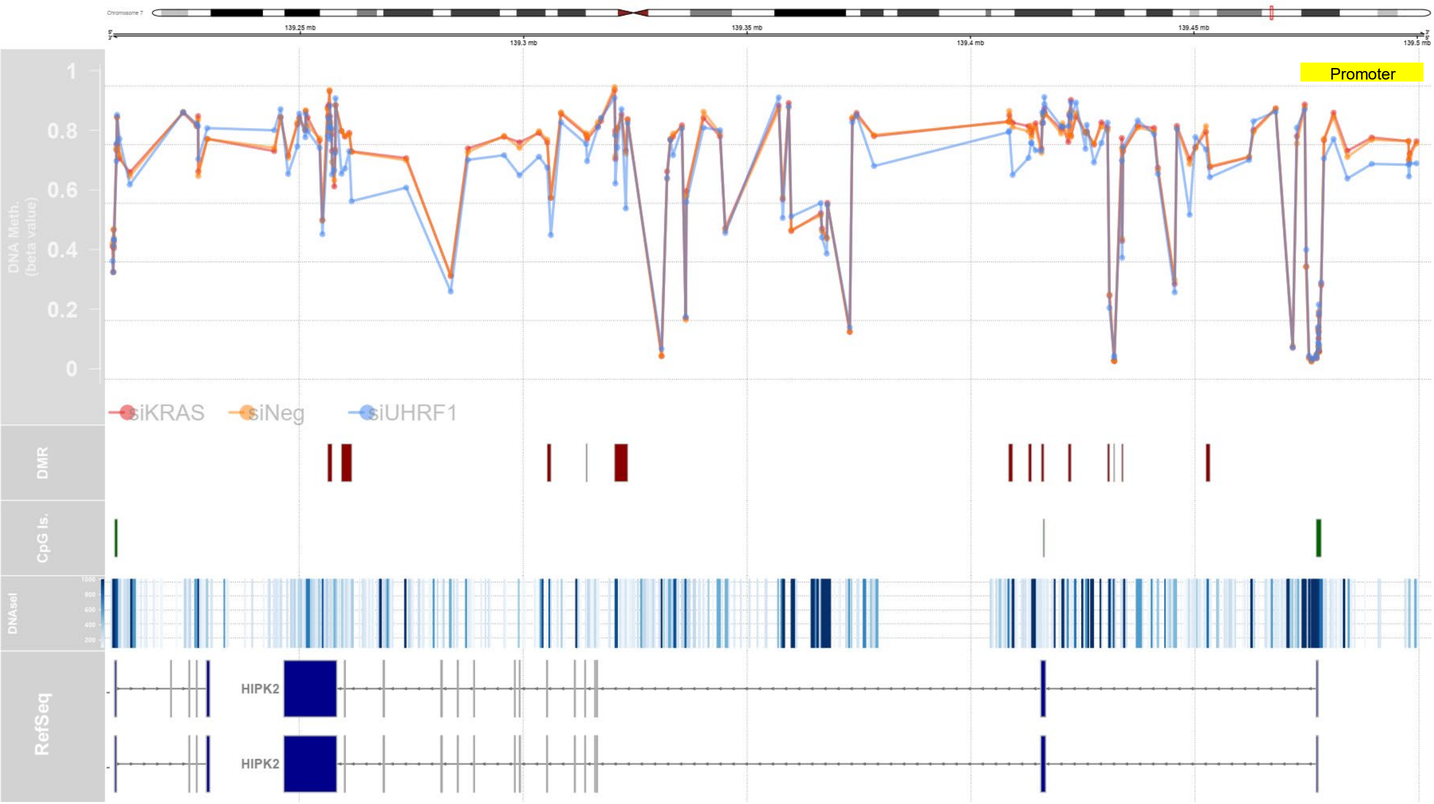

PTPN1

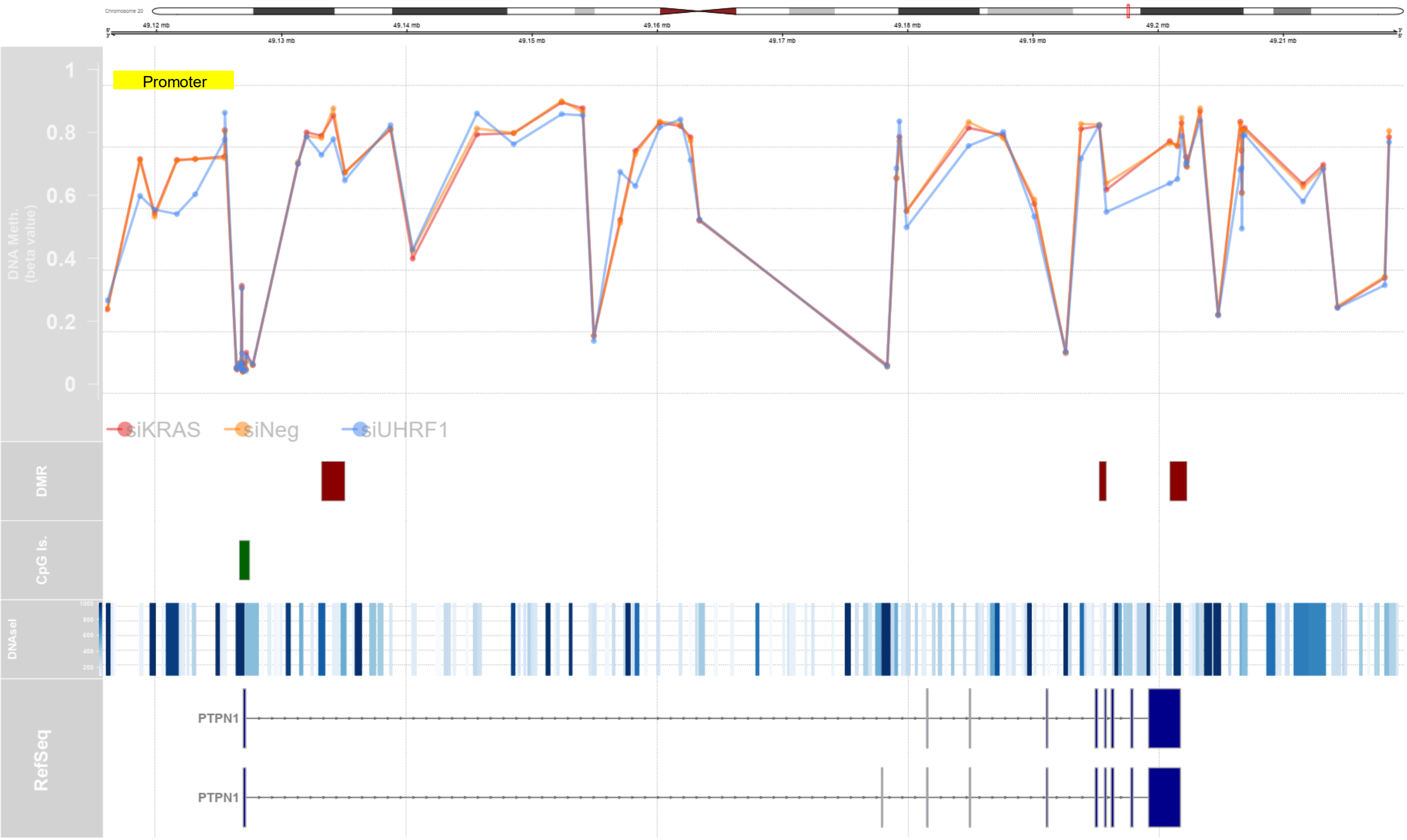

Supplementary Fig. 4

j WFDC1

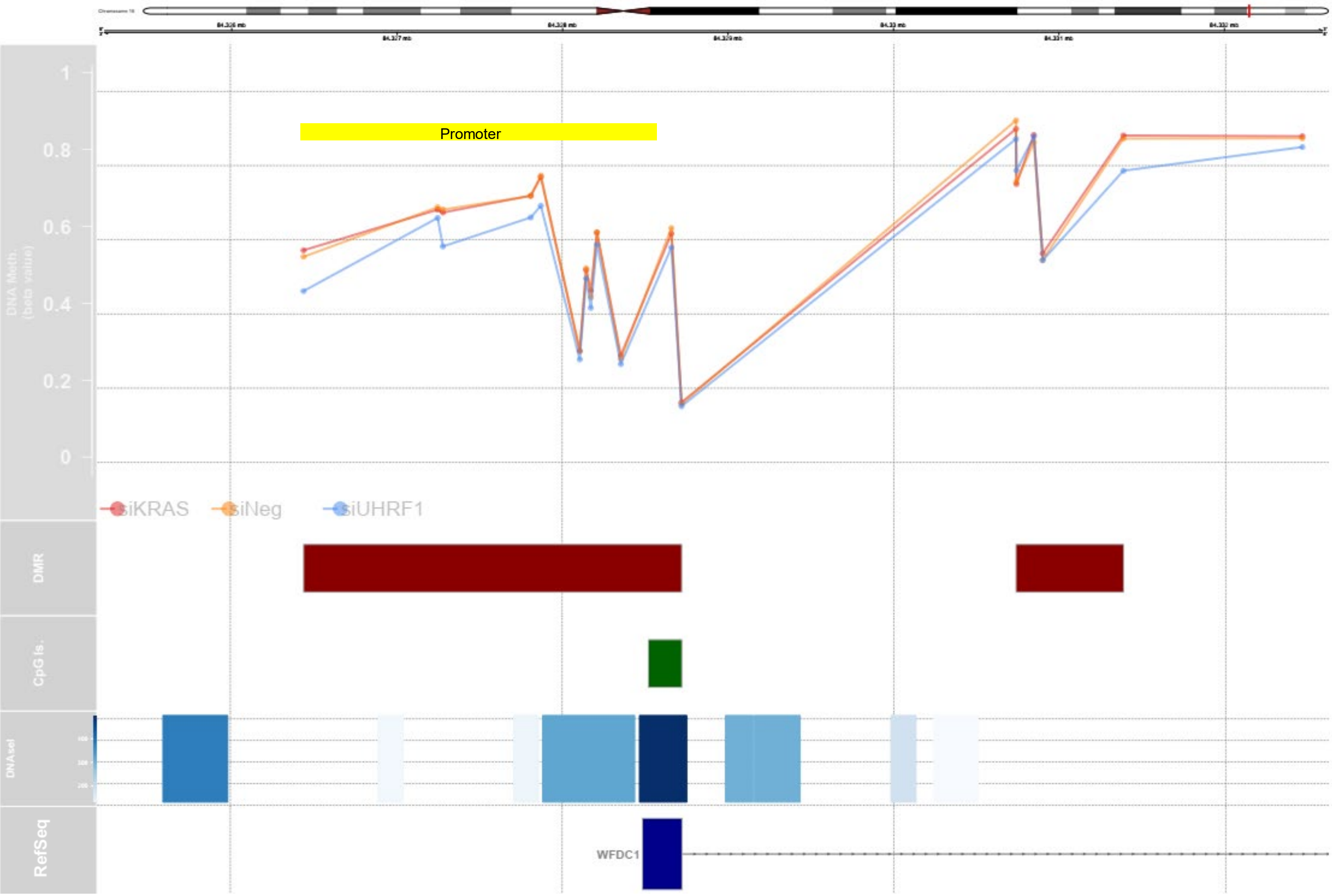

MFSD2A

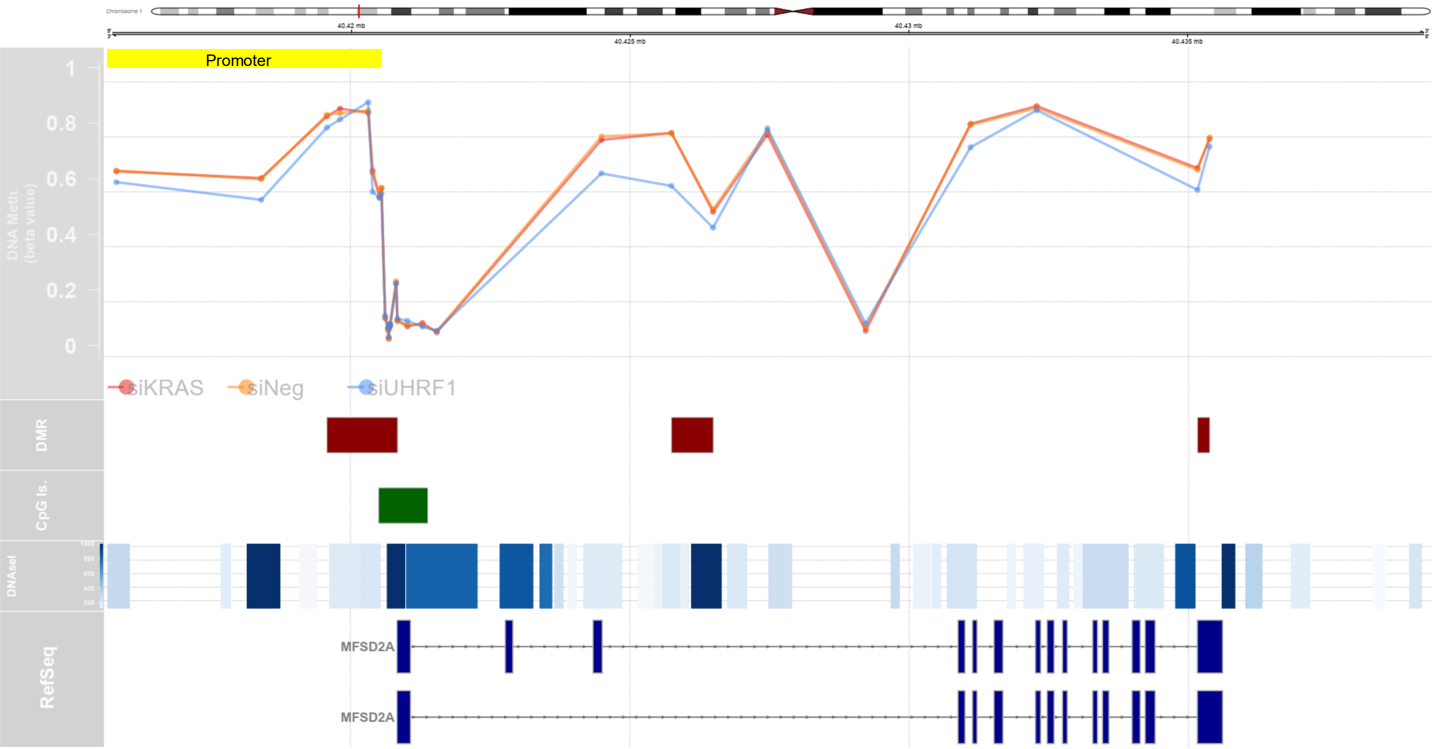

Supplementary Fig. 4

Supplementary Fig. 4. DNA methylation and gene expression analysis of UHRF1- or KRAS-depleted lung cancer cell lines. a, CpG profiling of two KRAS mutant lung cancer cell lines (A549, H358) treated with siRNAs against UHRF1, KRAS or a control negative siRNA (siNeg). Results shown in a two-dimensional tSNE plot of the top 10000 of most variable CpGs (on the basis of median absolute deviation). Each dot represents a sample. **b**, Global methylation trends. Left - the percentage of probes with significant log2-fold changes in methylation for siKRAS or siUHRF1 compared to siNeg control. Up/down group indicates higher or lower methylation in the treated group, respectively. Right plot - a percentage breakdown of significantly variable probes. Up/down indicates greater or lower variance in treated group, respectively. **c**, Pathway over-representation analysis (ORA) on Molecular Function GO terms of UHRF1-depleted cell lines. Pathways enriched in hypomethylated genes (“down”) indicated in yellow, pathways enriched in hypermethylated genes (“down”) indicated in blue. **d**, PCA analysis of differential gene expression of two KRAS mutant lung cancer cell lines (A549, H358) treated with siRNAs against UHRF1, KRAS or a control negative siRNA (siNeg). **e**, Volcano plot of differential gene expression in KRAS-depleted cells. Green points – examples of significantly overexpressed tumor suppressor genes (TSGs), blue point – UHRF1 and KRAS. **f**, Visualization of GSEA-based pathway analysis on differentially expressed genes in UHRF1-depleted cells. GSEA using gene sets from KEGG, WikiPathways, Biocarta, Hallmark and GO. Visualization using EnrichmentMap in Cytoscape. **g**, PROGENy analysis on RNAseq from two KRAS mutant cell lines (H358, A549) transfected with the indicated siRNAs. Pathways with significantly lower activity in siUHRF1 vs siNeg samples indicated with blue arrows, pathways with significantly higher activity indicated with red asterisks. **h**, GSEA on differentially expressed genes in UHRF1-depleted cells using a gene set of TSGs downregulated in lung adenocarcinoma (source: https://bioinfo.uth.edu/TSGene/). **i**, Venn diagram of tumor suppressor genes (TSGs) hypomethylated with siUHRF1 (EPIC methylation array dataset) and TSGs upregulated with siUHRF1 or siKRAS (RNAseq datasets) in A549 and H358 cells. Examples of common TSGs (n=66) are listed on the left. **j**, Graphical representation of four examples of TSGs (HIPK2, PTPN1, WFDC1, and MFSD2A) with decreased methylation in the promoter regions in UHRF1-depleted cells. The top layer corresponds to median beta values for each probe. DMR layer indicates the regions that were significantly different between siUHRF1 vs siNeg. CpG and DNAse I layers are annotation for predicated CpG island and sensitivity to DNAse I treatment, respectively. The bottom-most layer shows RefSeq transcript renderings.

Supplementary Fig. 5

a

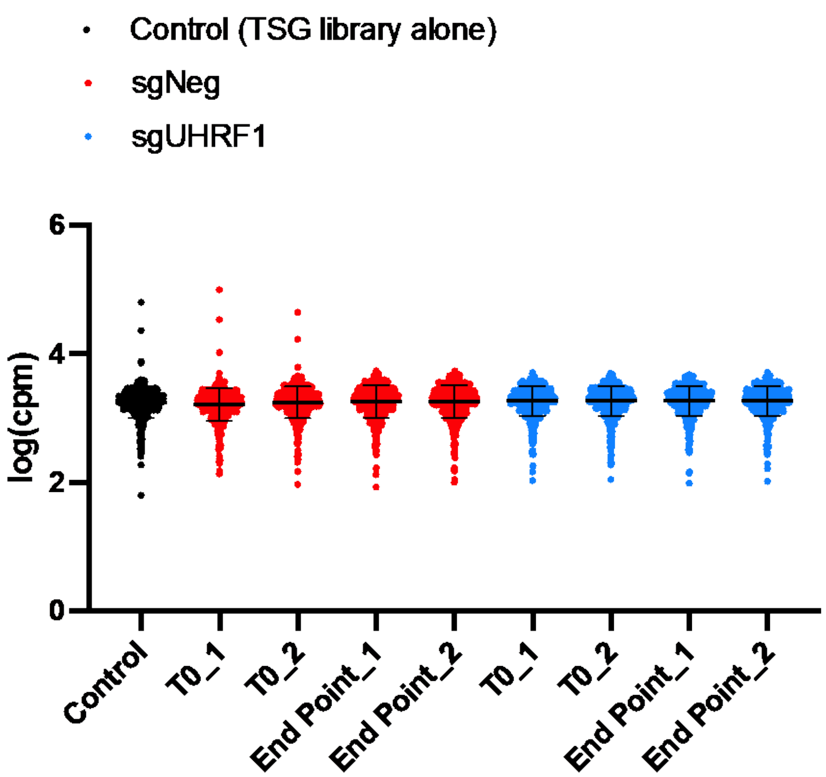

b

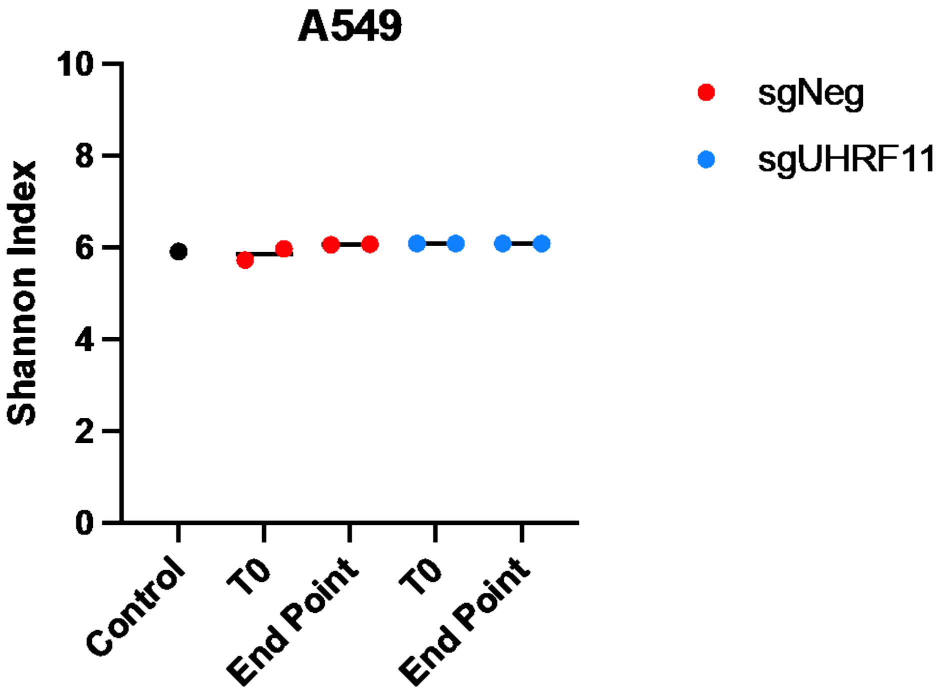

Supplementary Fig. 5

c

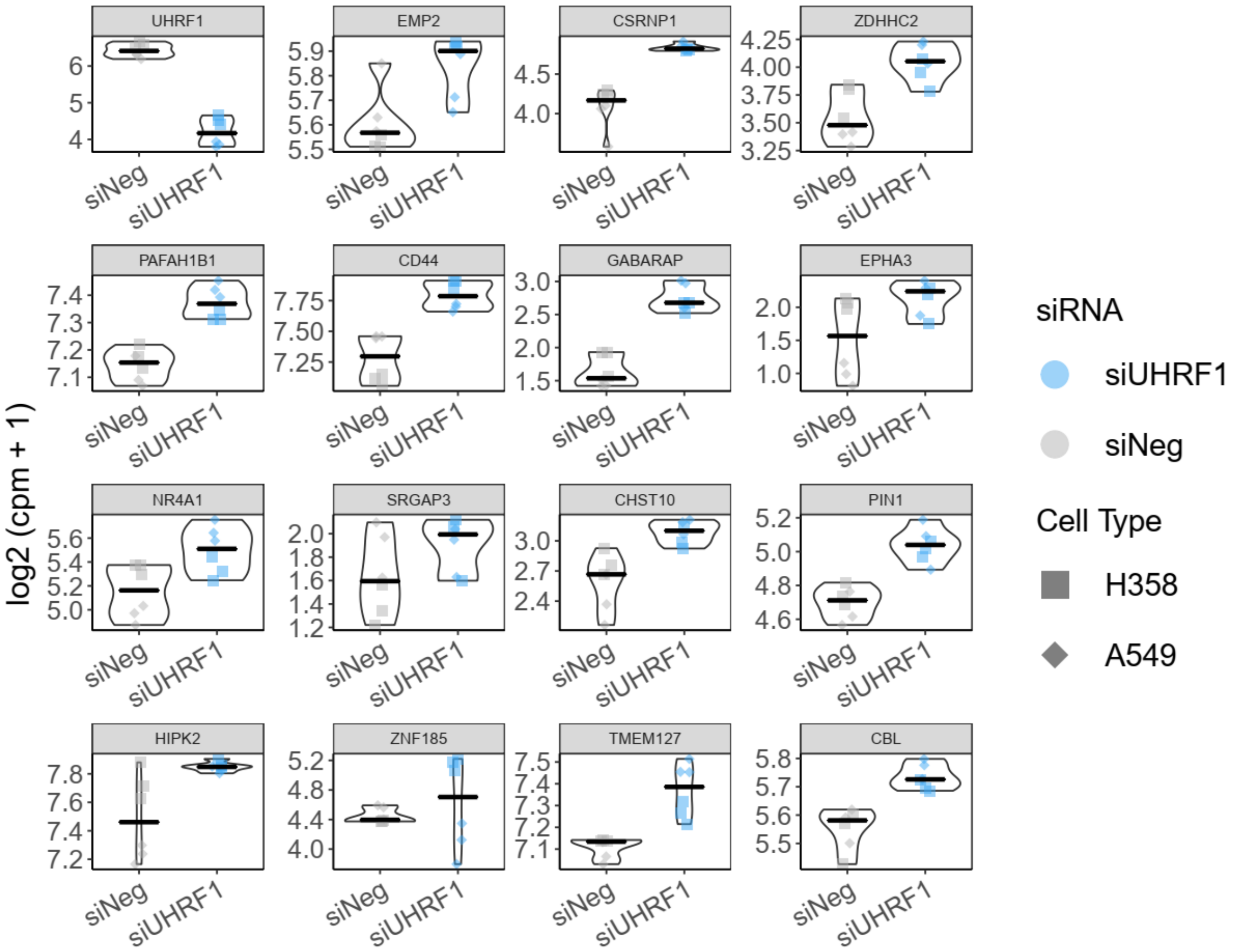

d

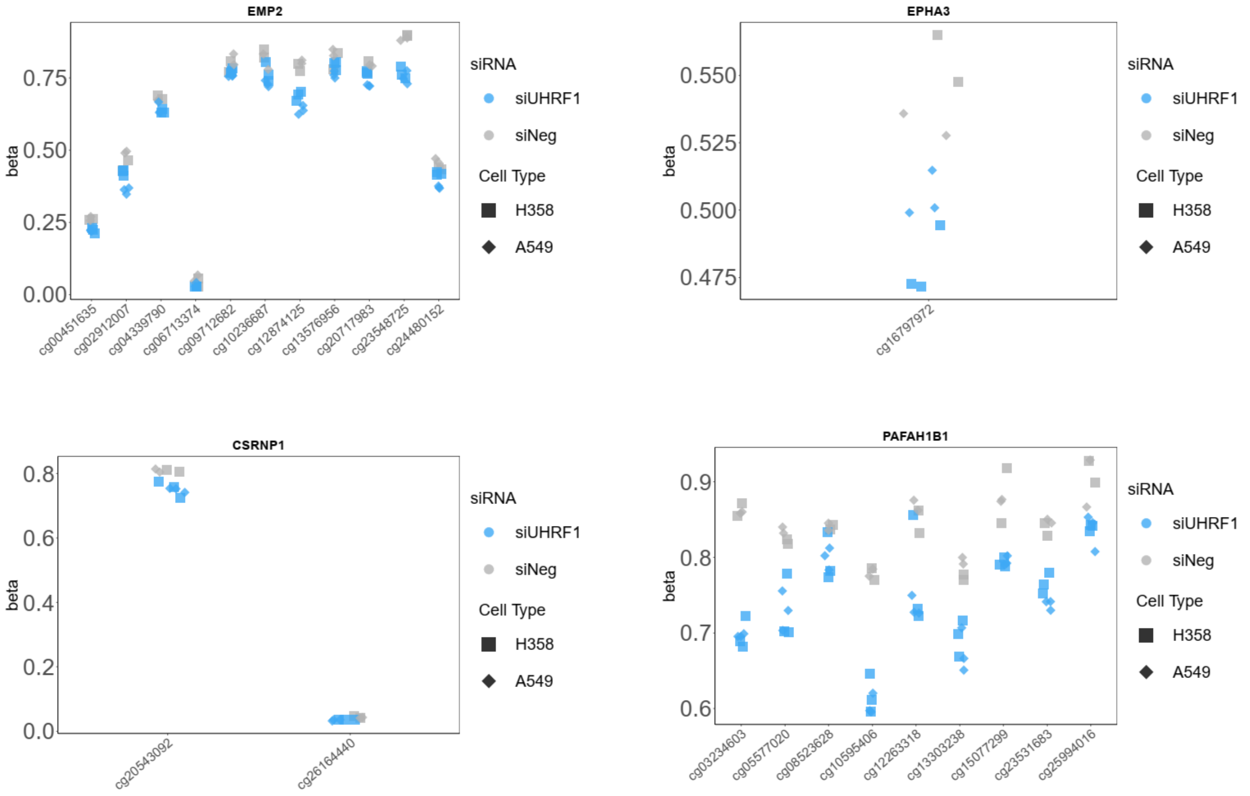

Supplementary Fig. 5

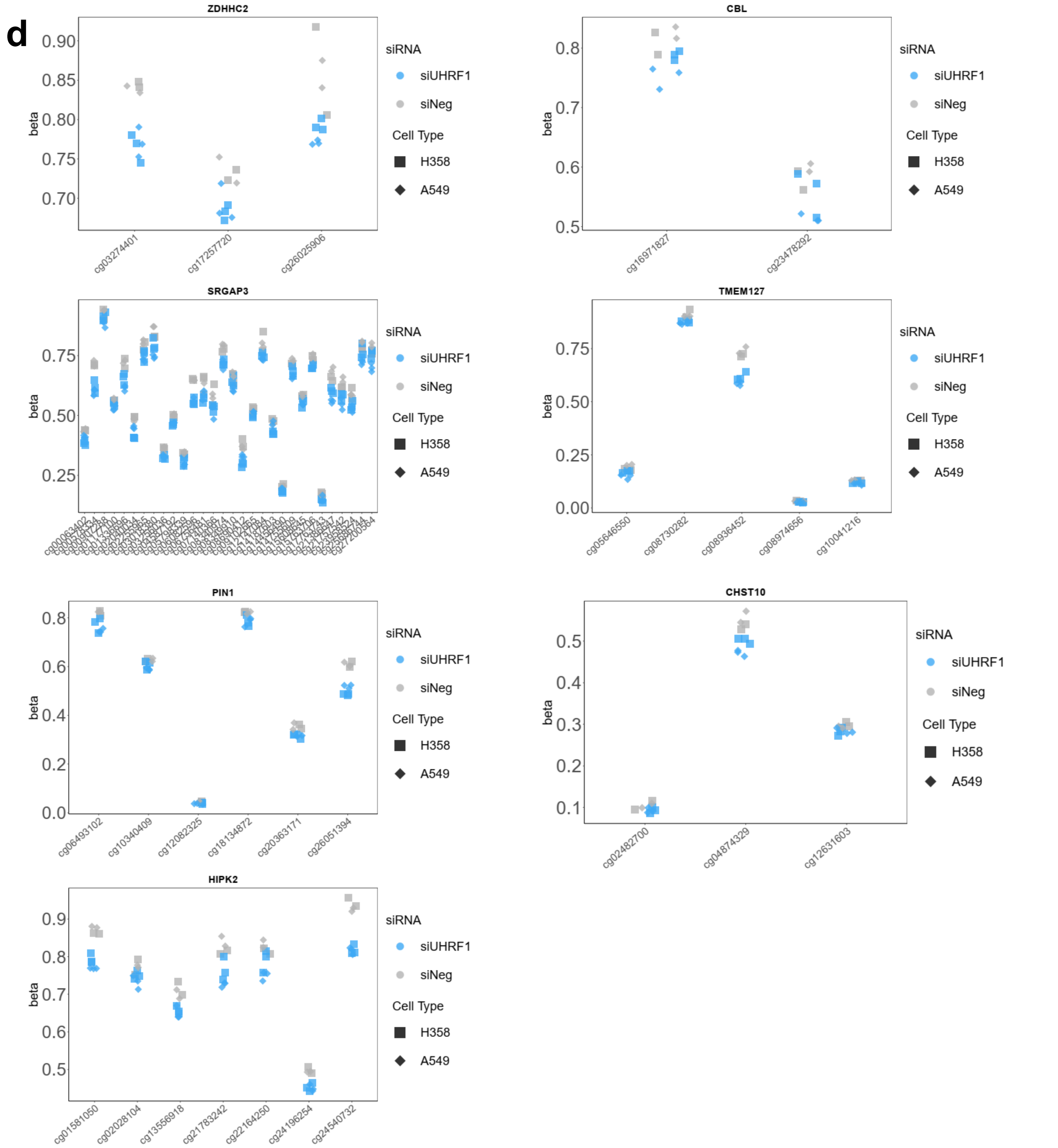

Supplementary Fig. 5. CRISPR minipool screen. a, Logarithmic representation of the average counts per million in A549-Cas9 cells transduced with the plasmid CRISPR library. T0 - cells after sgRNA (sgNeg or sgUHRF1) at day 0 (3 days post-transfection), End Point - cells at the end of the screen (18 days post-transfection), Control – cells containing the library but prior to sgRNA transfection. Horizontal lines represent mean values with one standard deviation error bars. **b,** Shannon index represented as scatter plot to show the diversity and complexity of recovered sgRNA pools in vitro at each screen time point. **c,** Expression of UHRF1 and 15 TSGs hits from the CRISPR screen in H358 and A549 cells (blue – siUHRF1, grey – siNeg). The y-axis shows log2(cpm+1). **d**, Probes with statistically significant hypomethylation (adjusted p-value < 0.05) in regions related to the promoter (annotated as promoter-associated, 5' UTR, 3' UTR, TSS, and/or related to the 1st exon of the gene). The y-axis represents beta values color-coded by siRNA treatment (blue – siUHRF1, grey – siNeg). Source data are provided as a Source data file.

Supplementary Fig. 6

a

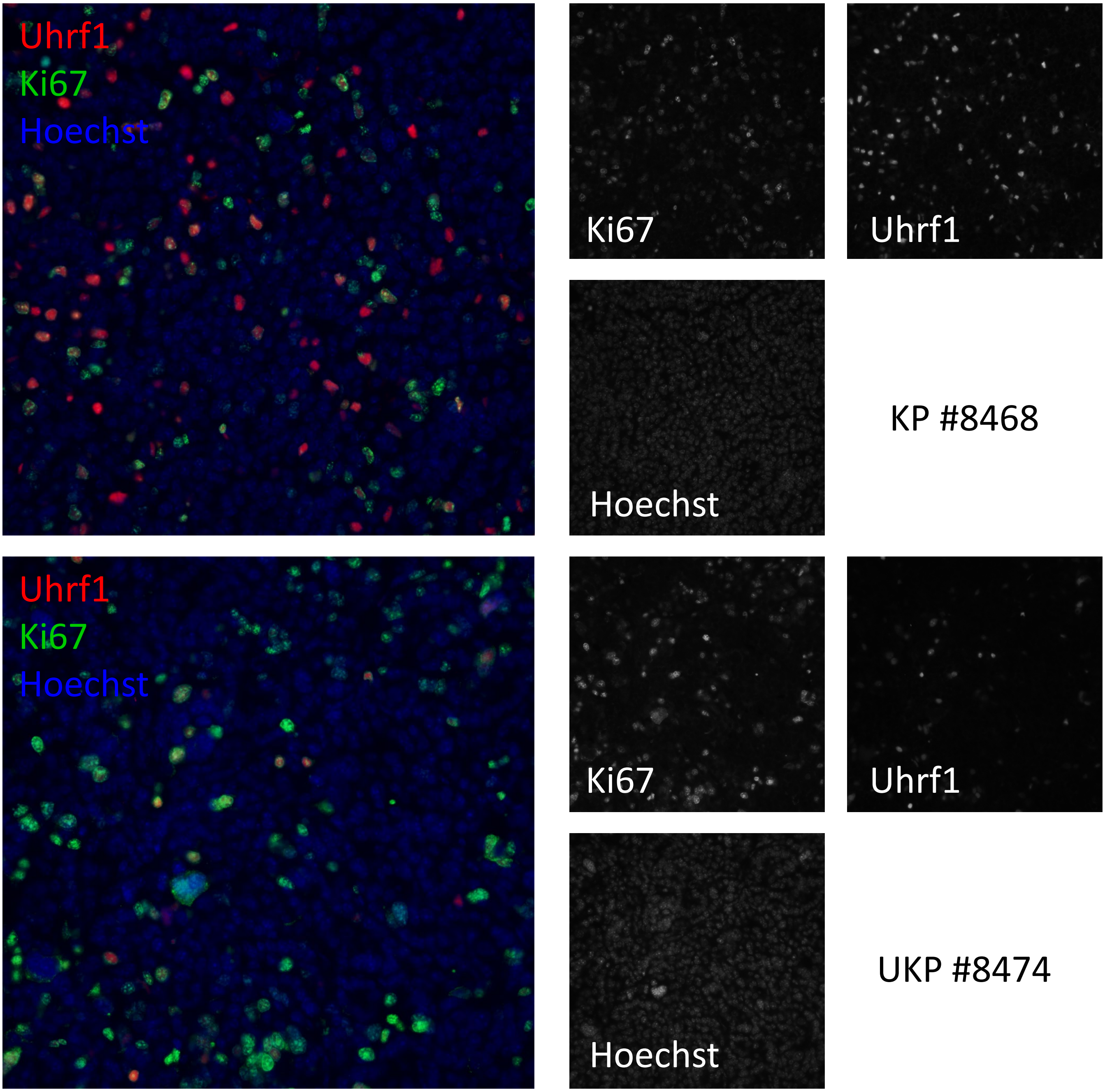

b

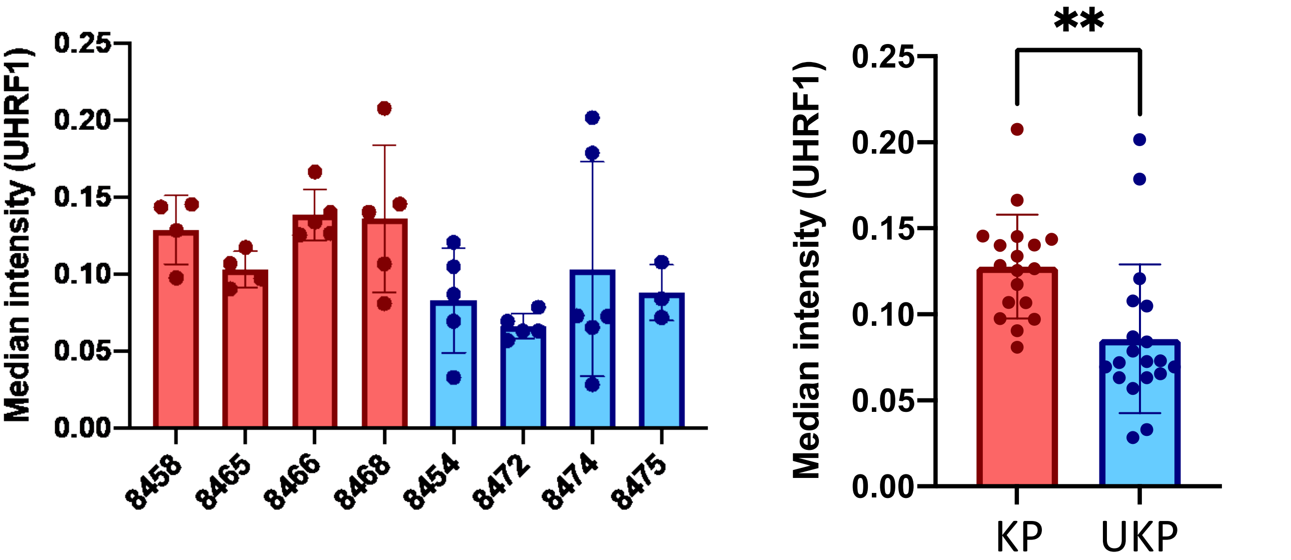

Supplementary Fig. 6

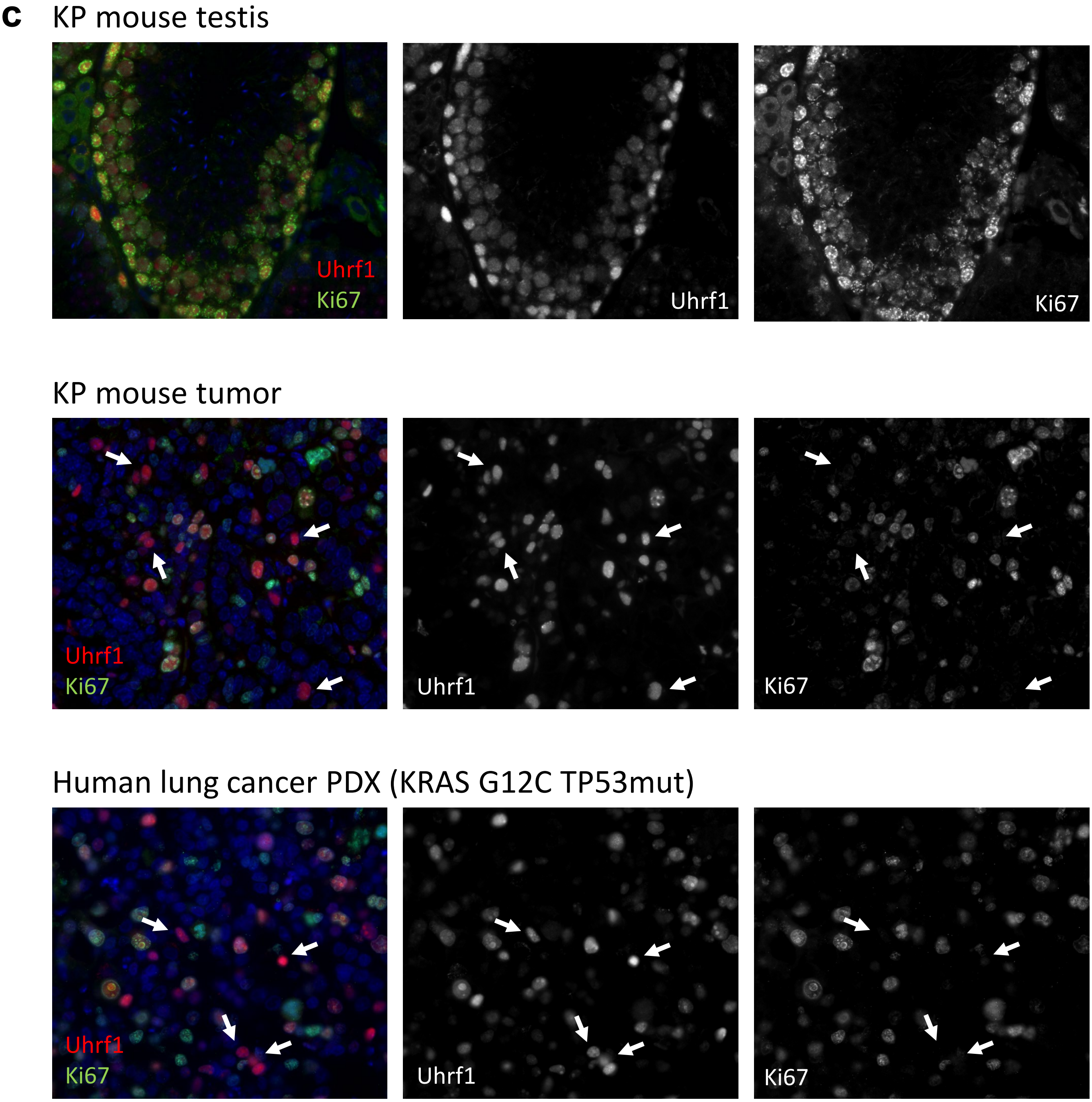

Supplementary Fig. 5

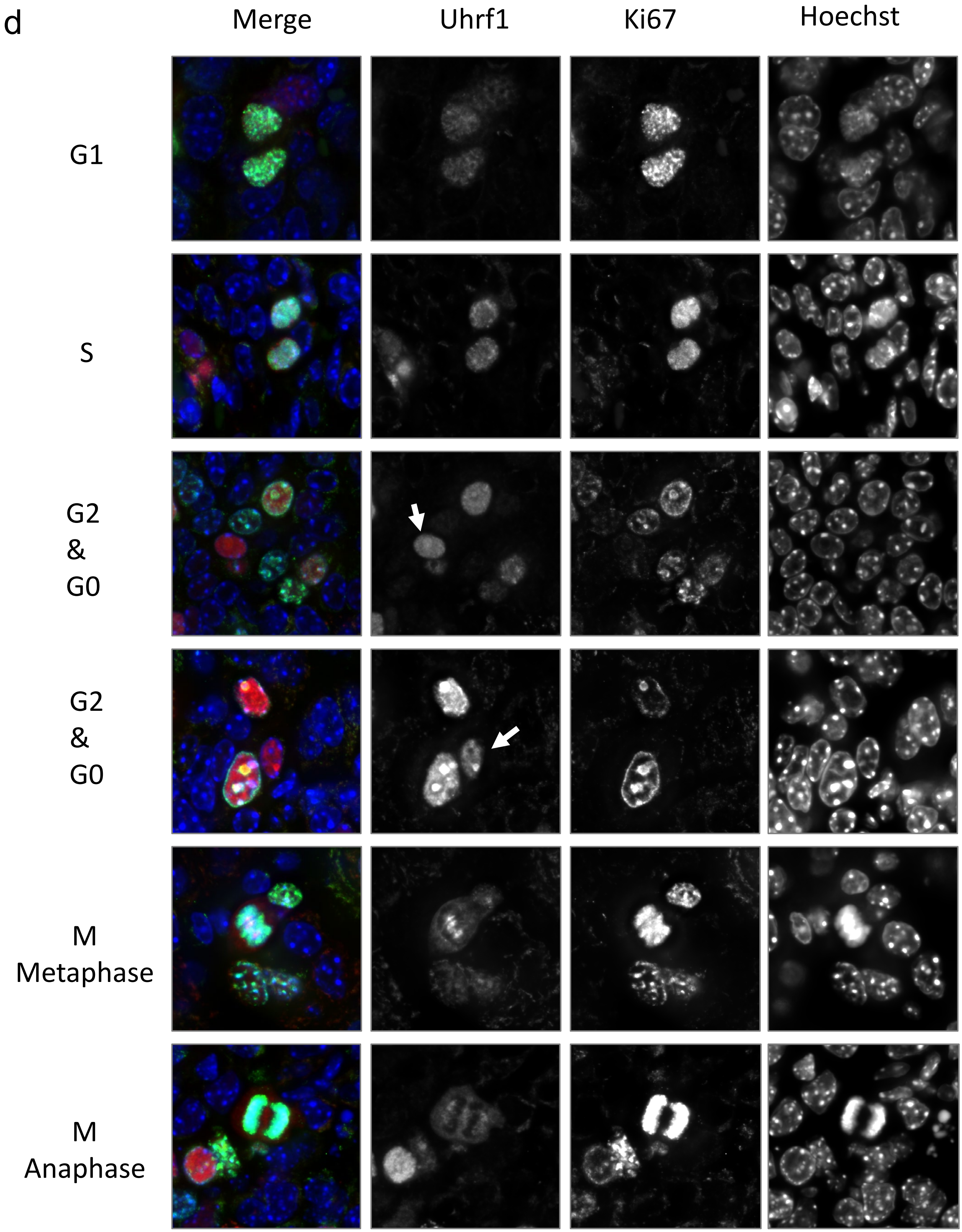

Supplementary Fig. 6

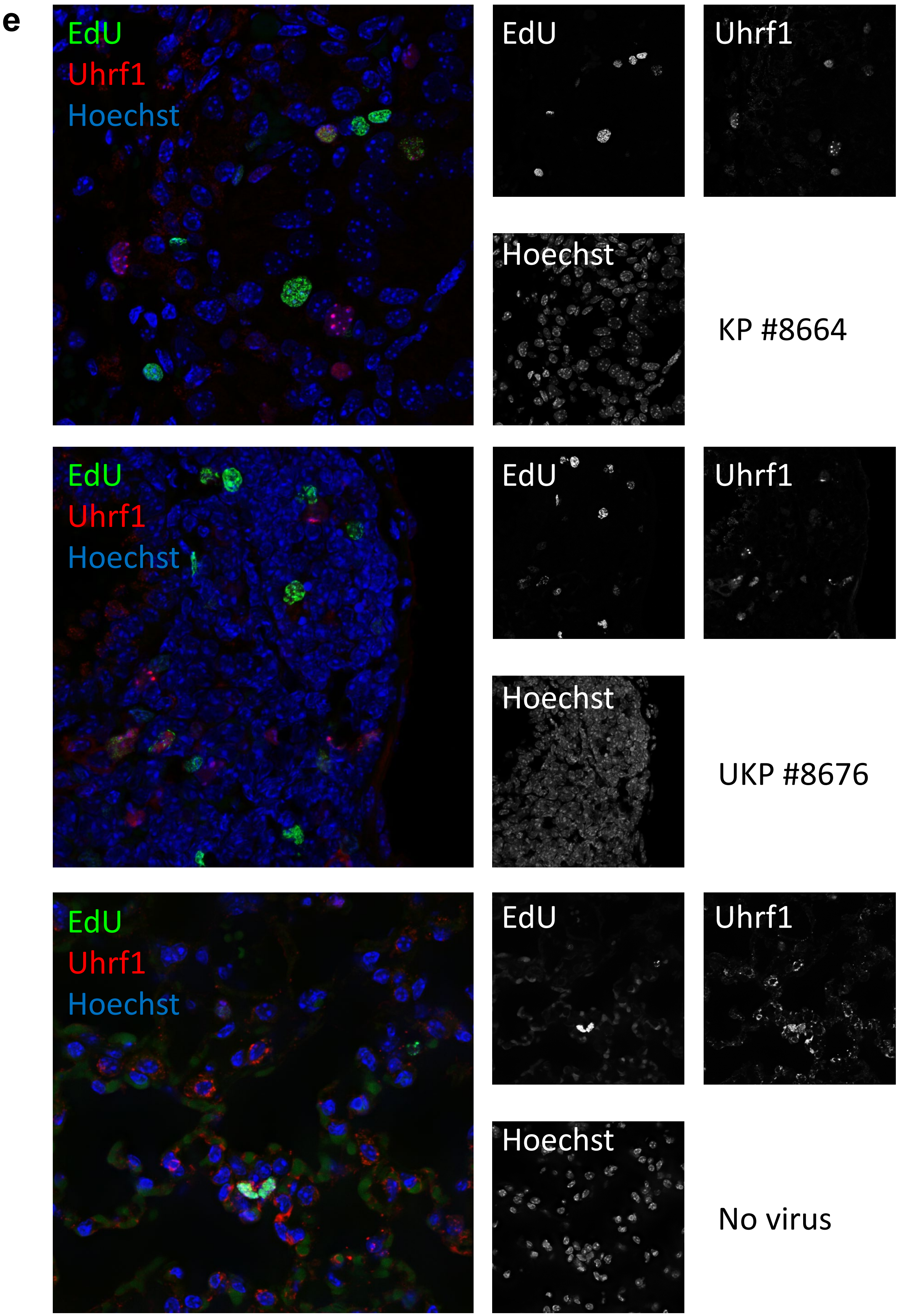

Supplementary Fig. 6

f

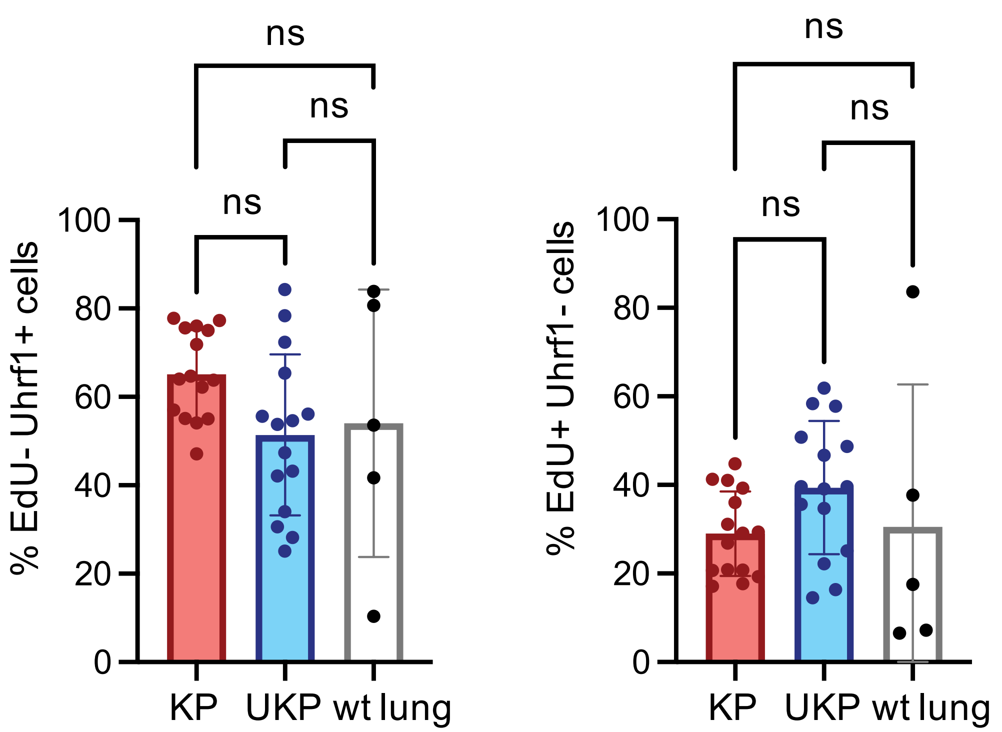

g

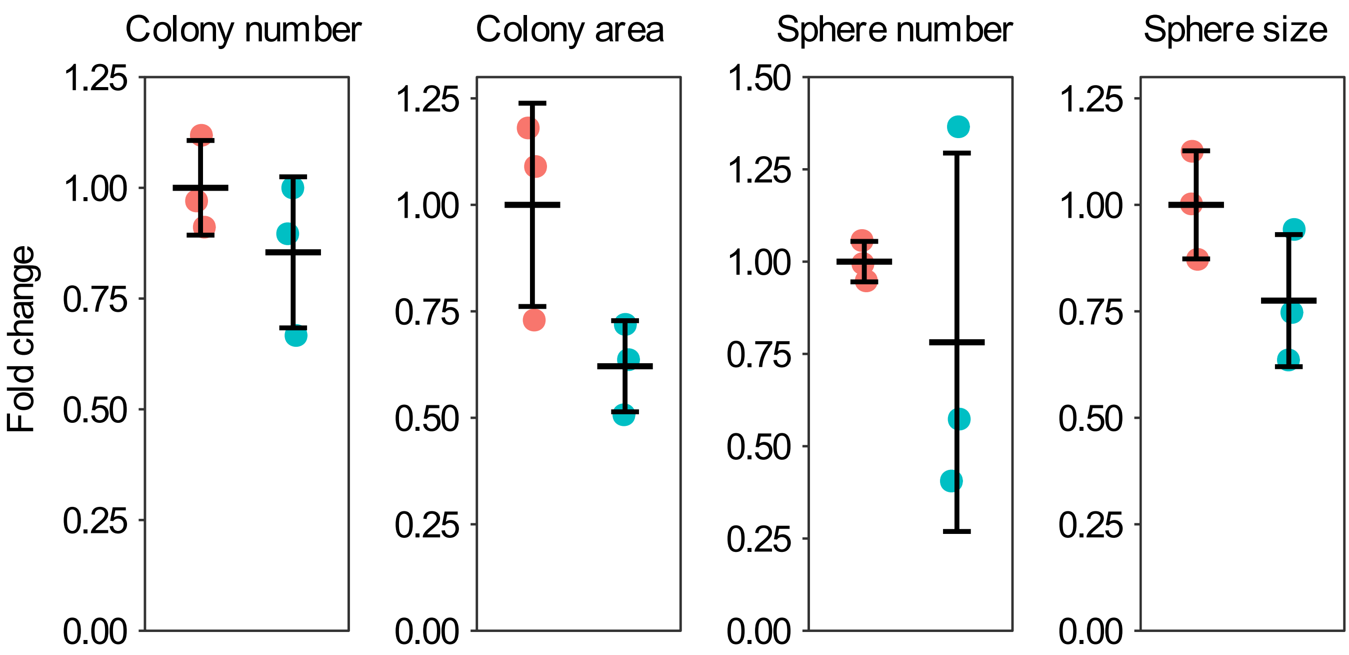

Supplementary Fig. 6

Supplementary Fig. 6. UHRF1 protein expression in KP and UKP tumors. **a**, Representative images of immunofluorescence imaging of paraffin-embedded sections of tumor-bearing lungs from a KP (top) and a UKP (bottom) mouse. Lungs collected 16 weeks post adeno-Cre infection. Sections immunostained with the indicated antibodies and imaged using a 40x objective and a monochrome camera with the same exposure settings. **b**, Left - Quantification of fluorescence intensity in the green channel (UHRF1 immunostaining) from 4 KP mice (red bars) and 4 UKP mice (blue bars); n = 3-6 images per mouse. Right – Cumulative data from the plot on the left; n=18 images for KP and n=19 images for UKP mice. Bars represent means; ** p = 0.0016 by unpaired two-sided Student's t-test. **c**, Immunofluorescence imaging of paraffin-embedded sections of normal mouse testis from an untreated KP mouse (top), tumor-bearing lungs from a KP mouse 16 weeks post-Cre infection (middle) and a human lung cancer patient-derived xenograft (PDX) model (bottom). Sections co-stained with anti-UHRF1 and anti-Ki67 antibodies and Hoechst nuclear dye. Images acquired using a 40x objective. Exposure settings were not conserved between images. White arrows point to UHRF1-positive Ki67-negative nuclei in tumor sections. Representative images of n=5 individual images. **d**, High resolution immunofluorescence imaging of paraffin-embedded sections of tumor-bearing lungs from a KP mouse. Immunostaining indicated above the images. White arrows point to nuclei presumed to be in the G0 phase of the cell cycle. Images acquired using a 100x oil objective using the same exposure settings for all images. Images derived from a series of n=5 individual images. **e**, Immunofluorescence imaging of paraffin-embedded lung sections from mice treated with EdU for 16h. KP, UKP – mice 11 weeks post adeno-Cre, No Virus – control untreated mouse. Images acquired using a 100x oil objective using the same exposure settings for all images. Representative images of n=5 individual images are shown. **f**, Quantification of Uhrf1+EdU- cells (left) and EdU+Uhrf1- cells (right) in IF images from mice treated with EdU. Number of individual images quantified: n=15 (KP and UKP), n=5 (control no-virus mouse). Bars represent means with with standard deviation error bars; ns - not significant by Kruskal-Wallis test. **g**, Quantification of 2D colony growth (two left plots) and 3D sphere growth (two right plots) of UKP cells treated with empty adenovirus (red points) or Cre-expressing adenovirus (blue points). Data represented as means of 3 technical replicates with standard deviation error bars. Source data are provided as a Source data file.

Supplementary Fig. 7

a

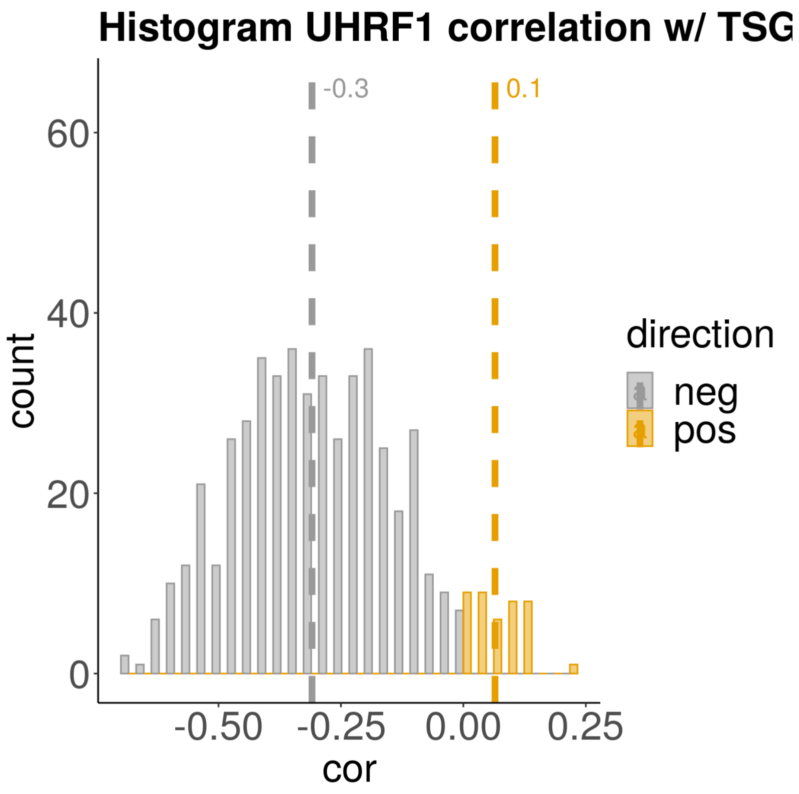

b

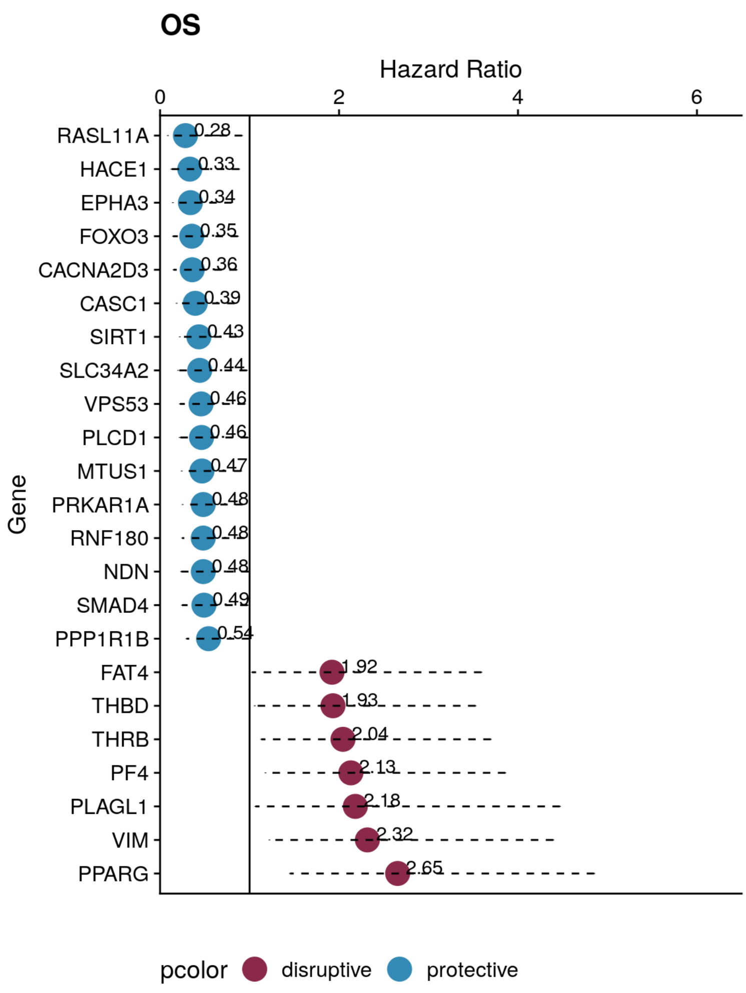

Supplementary Fig. 7

c

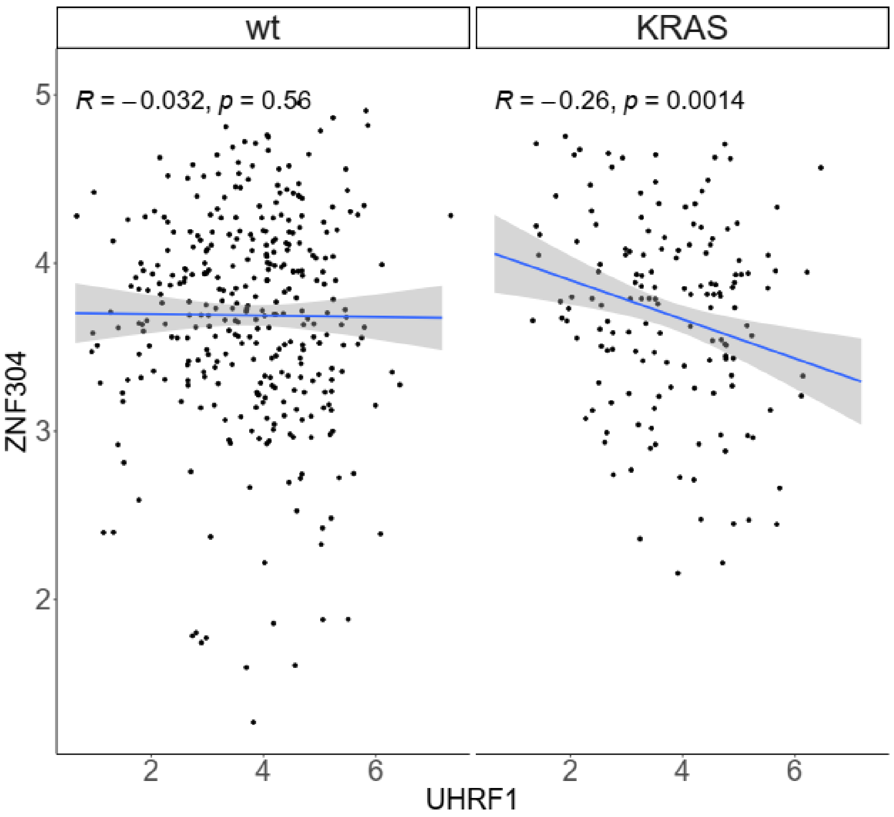

d

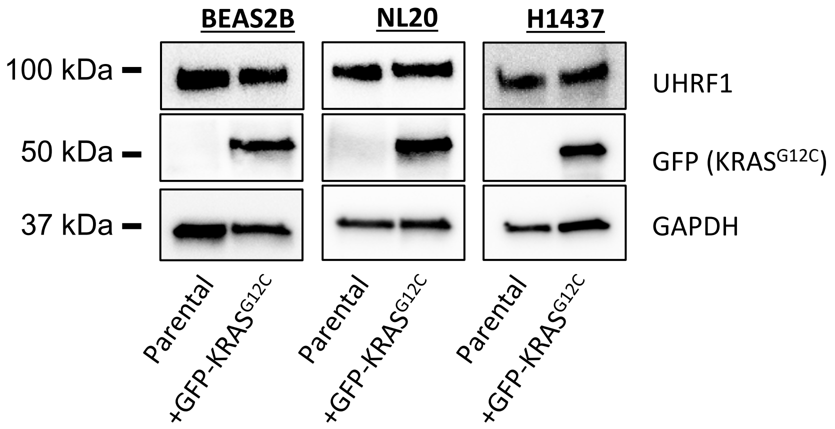

Supplementary Fig. 7. **a**, Histogram of UHRF1 correlation with TSG expression in LUAD. Neg indicates anti-correlation between UHRF1 gene expression and the TSG gene expression, pos indicates positive correlation. Vertical dotted lines represent the average correlation coefficient. **b**, Plot representing genes whose expression is anticorrelated with UHRF1 and KRAS in the LUAD dataset and in addition is protective (HR < 1) specifically in KRAS mutant LUAD samples. HR – hazard ration. **c**, Correlation of UHRF1 and ZNF304 expression (log2CPM) in KRAS mutant and KRAS wild type subsets of the LUAD dataset (TCGA). Correlation coefficient and p-value computed using Pearson's product-moment correlation test. Linear trend line was generated using a linear model, shaded confidence region represents CI=0.95. **d**, Western blot for UHRF1, GFP and GAPDH in 3 KRAS wild-type cell lines (BEAS2B, NL20 and H1437) transfected with GFP-tagged KRAS G12C construct. Extracts collected 4 days post transient transfection. Experiment was performed once.

Supplementary Fig. 3a
Chemidoc images

Uhrf1 (sc373750) 1:100

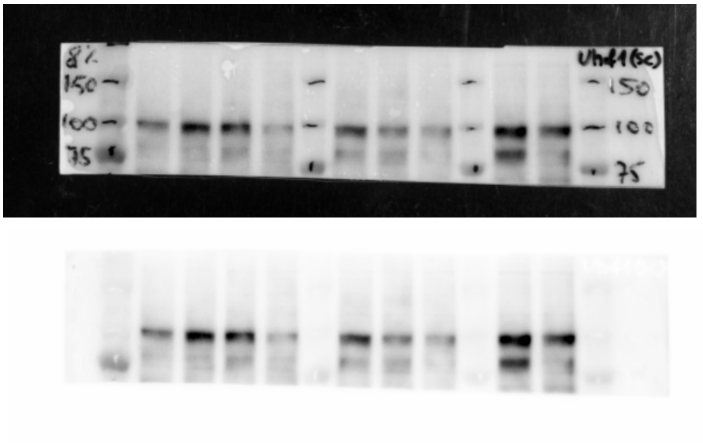

Sample order:
1. protein ladder
2. H358
3. H2009
4. H23
5. A549
6. protein ladder
7. H1437
8. H1568
9. H1650
10. protein ladder
11-12. (unrelated samples)

Actin (A5316) 1:5000

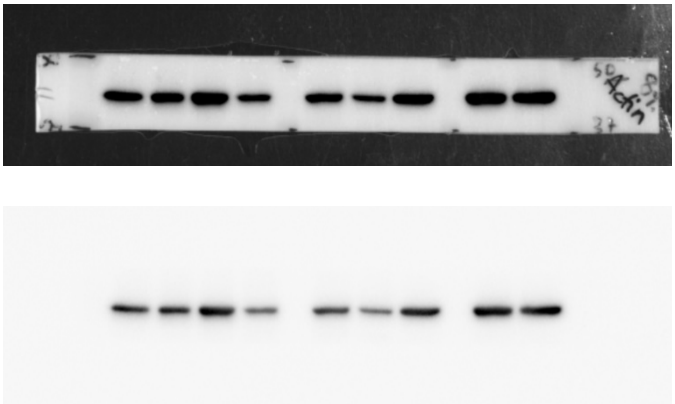

Supplementary Fig. 3c
Chemidoc images

Sample order:
1. protein ladder
2. H2009 siNeg
3. H2009 siKRAS
4. H2009 siUHRF1
5. H358 siNeg
6. H358 siKRAS
7. H358 siUHRF1
8. (empty)
9. H23 si Neg
10. H23 siKRAS
11. H23 siUHRF1
12. A549 siNeg
13. A549 siKRAS
14. A549 siUHRF1
15. (empty)
16. H1437 siNeg
17. H1437 siKRAS
18. H1437 siUHRF1
19. H1568 siNeg
20. H1568 siKRAS
21. H1568 siUHRF1
22. (empty)
23. H1650 siNeg
24. H1560 siKRAS
24. H1560 siUHRF1
25. protein ladder

UHRF1 (ab213223) 1:500

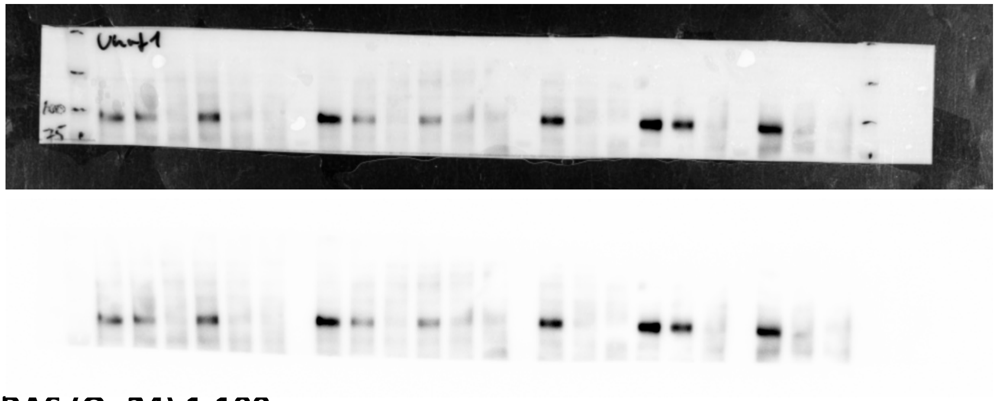

KRAS (Op24) 1:100

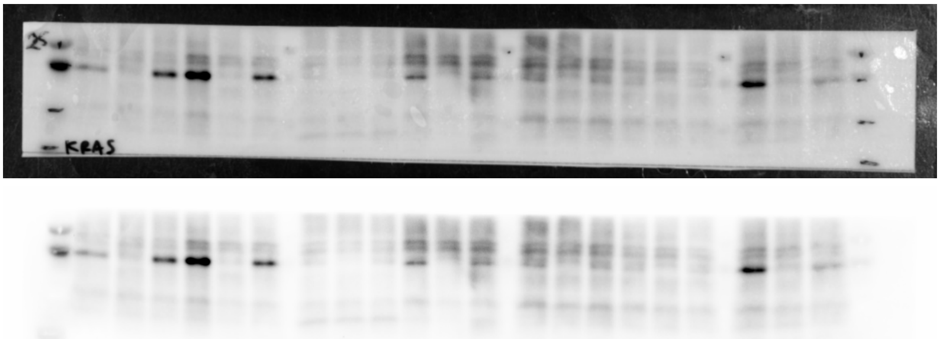

Cyclin B1 (ab32053) 1:5000

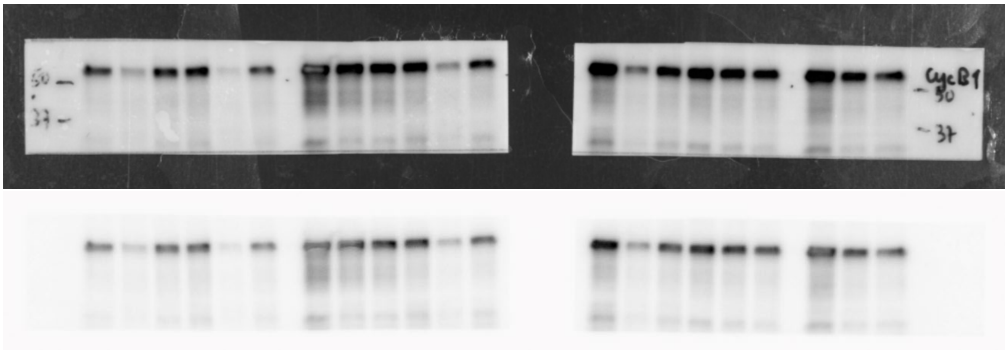

GAPDH (ab9485) 1:5000

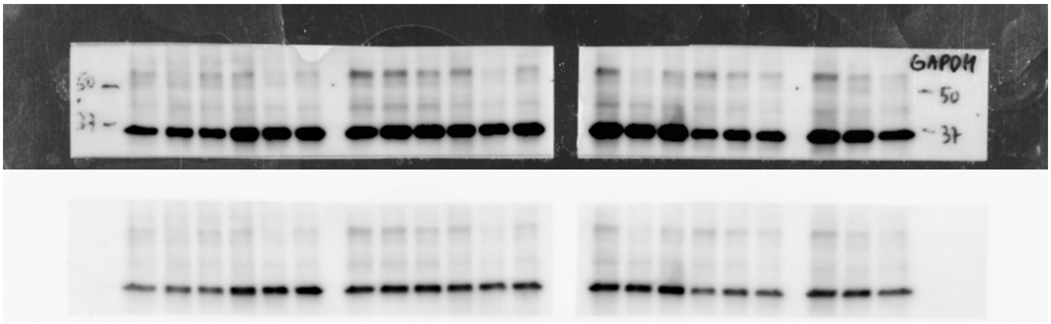

Supplementary Fig. 3d
Chemidoc images

Uhrf1 (sc373750) 1:100

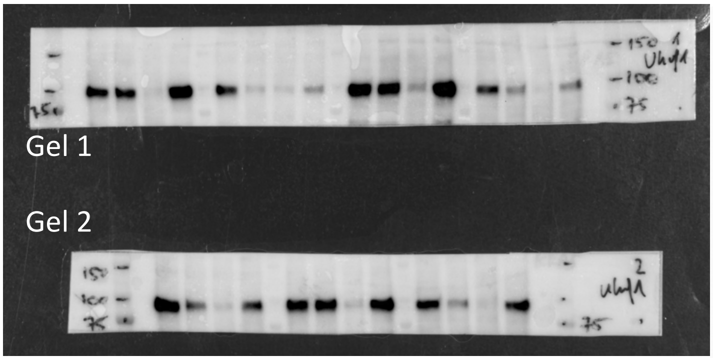

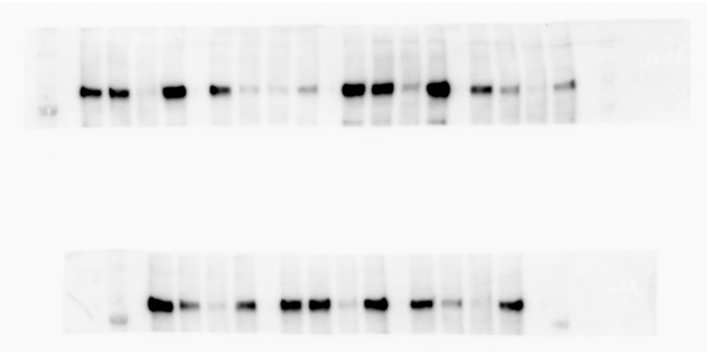

Cyclin D1 (ab134175) 1:5000

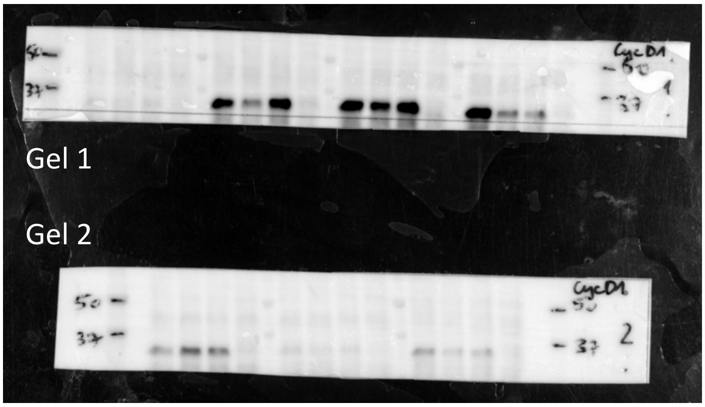

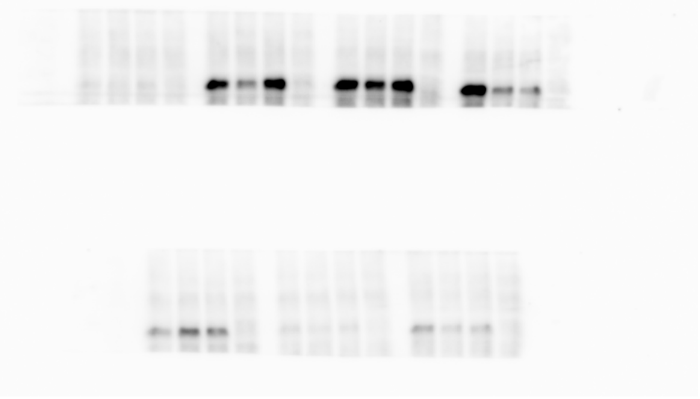

KRAS (WH0003845M1) 1:100

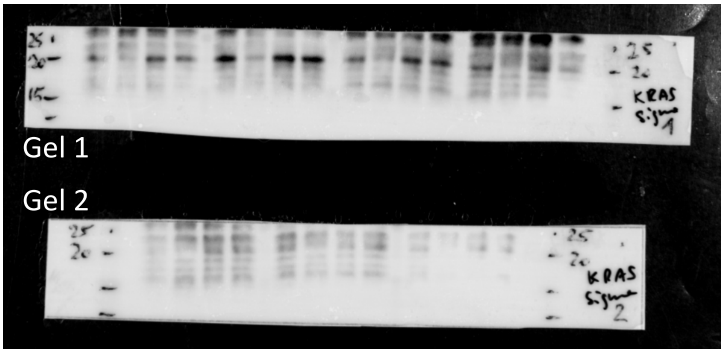

short exposure (5s)

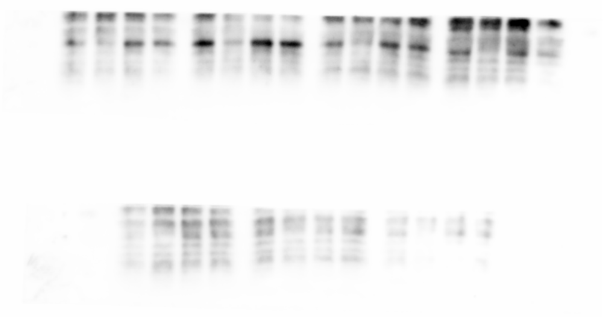

long exposure (20s)

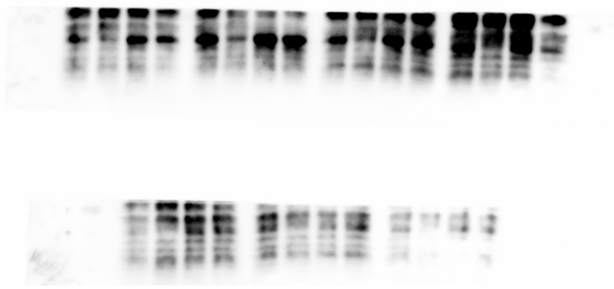

Supplementary Fig. 3d
Chemidoc images

GAPDH (ab9485) 1:5000

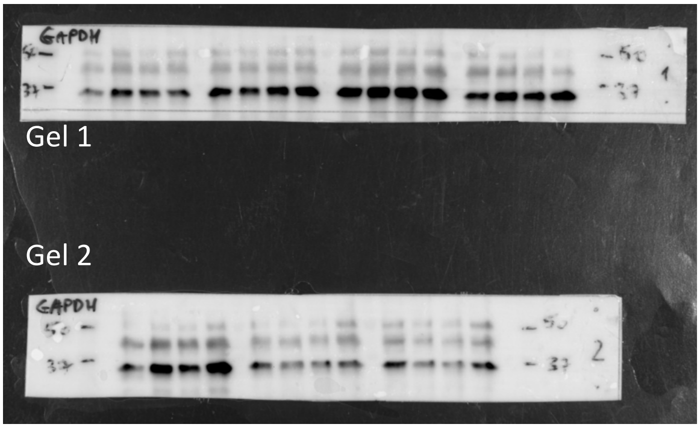

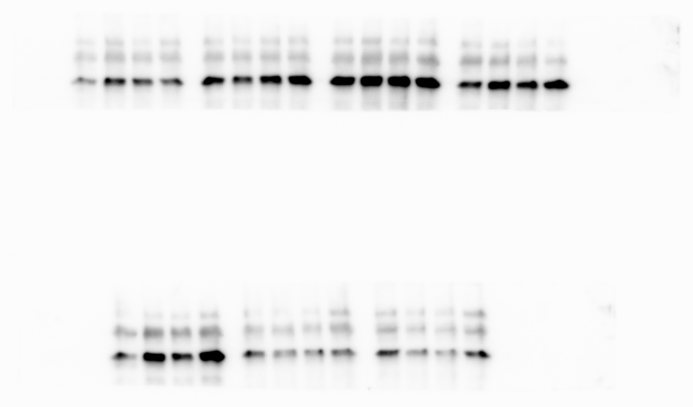

Sample order - gel 1
1. protein ladder
2. (empty)
3. H2009 siNeg
4. H2009 siKRAS
5. H2009 siUHRF1
6. H2009 siCyclinD1
7. protein ladder
8. H358 siNeg
9. H358 siKRAS
10. H358 siUHRF1
11. H358 siCyclinD1
12. protein ladder
13. H23 siNeg
14. H23 siKRAS
15. H23 siUHRF1
16. H23 siCyclinD1
17. protein ladder
18. A549 siNeg
19. A549 siKRAS
20. A549 siUHRF1
21. A549 siCyclinD1
22. (empty)
23. protein ladder

Sample order - gel 1
1. protein ladder
2. (empty)
3. H1437 siNeg
4. H1437 siKRAS
5. H1437 siUHRF1
6. H1437 siCyclinD1
7. protein ladder
8. H1568 siNeg
9. H1568 siKRAS
10. H1568 siUHRF1
11. H1568 siCyclinD1
12. protein ladder
13. H1650 siNeg
14. H1650 siKRAS
15. H1650 siUHRF1
16. H1560 siCyclinD1
17. (empty)
18. protein ladder

Supplementary Fig. 3f
Chemidoc images

pERK (#4370) 1:1000

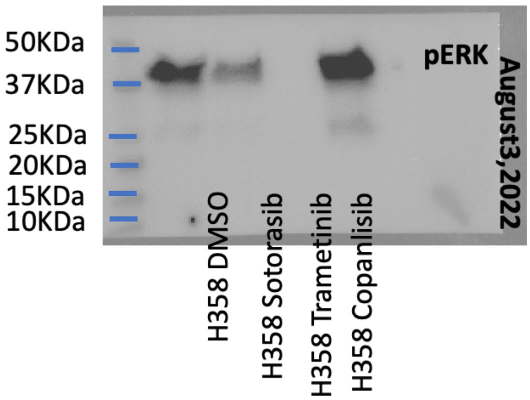

ERK (#4795) 1:1000

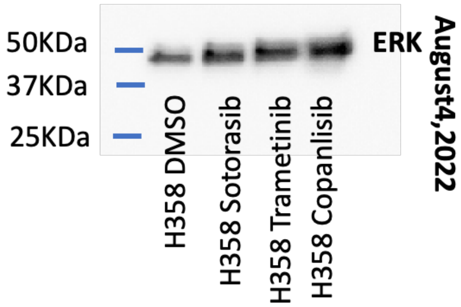

pAKT (#13038) 1:1000

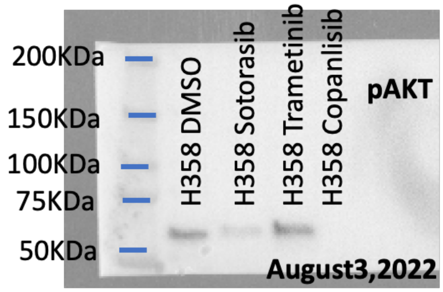

AKT (#75692) 1:1000

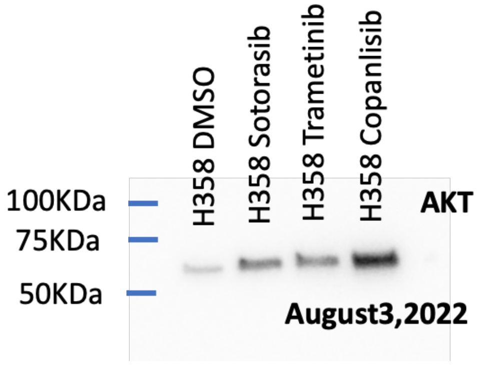

Uhrf1 (sc373750) 1:100

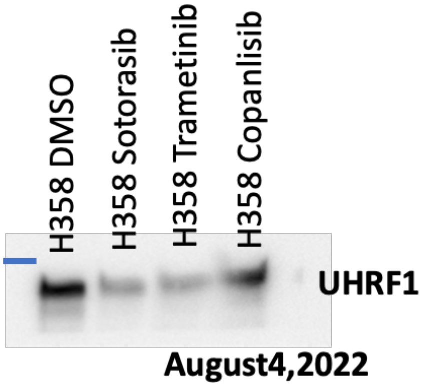

CCND1 (ab134175) 1:10000

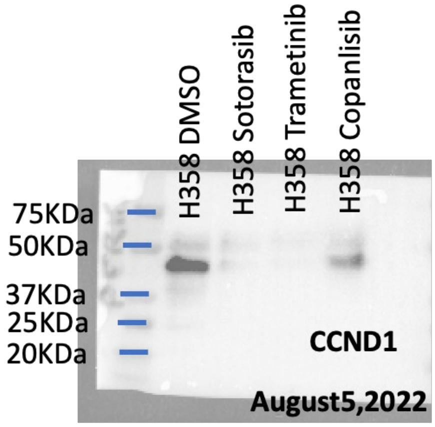

GAPDH (ab9485) 1:5000

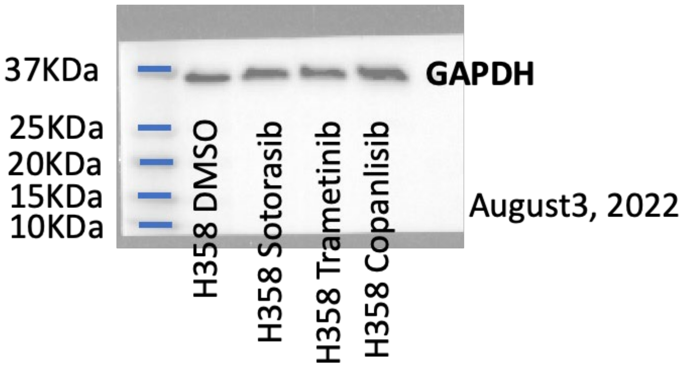

Supplementary Fig. 3g
Chemidoc images

Uhrf1 (sc373750) 1:100

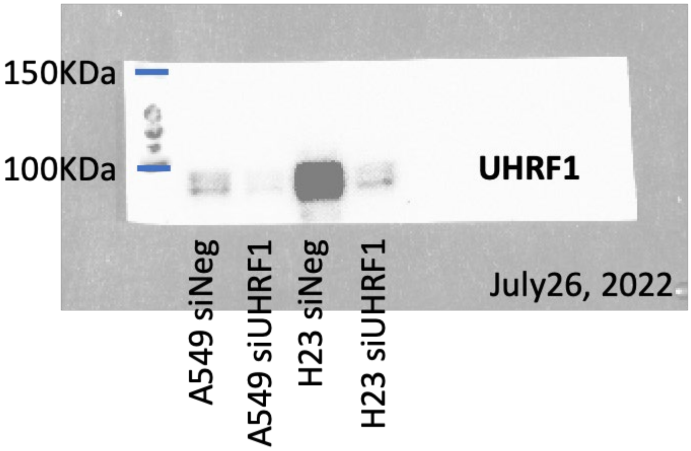

Short exposure

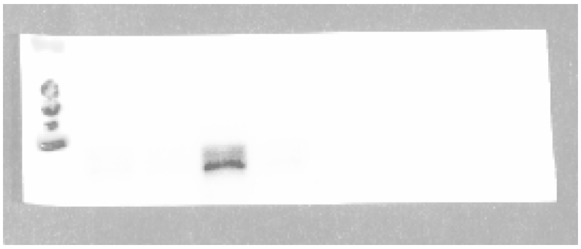

Tubulin (#2148) 1:1000

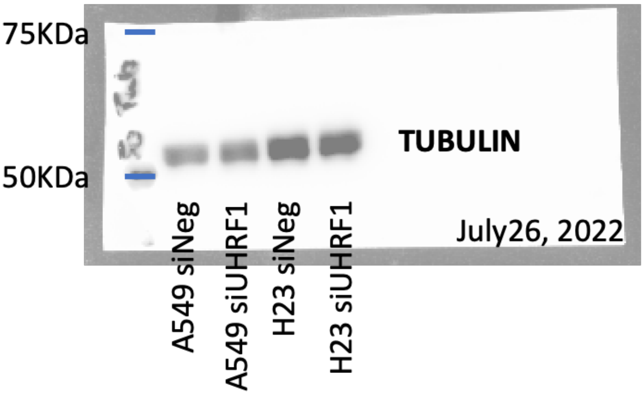

Supplementary Fig. 7d
Chemidoc images

Uhrf1 (sc373750) 1:100

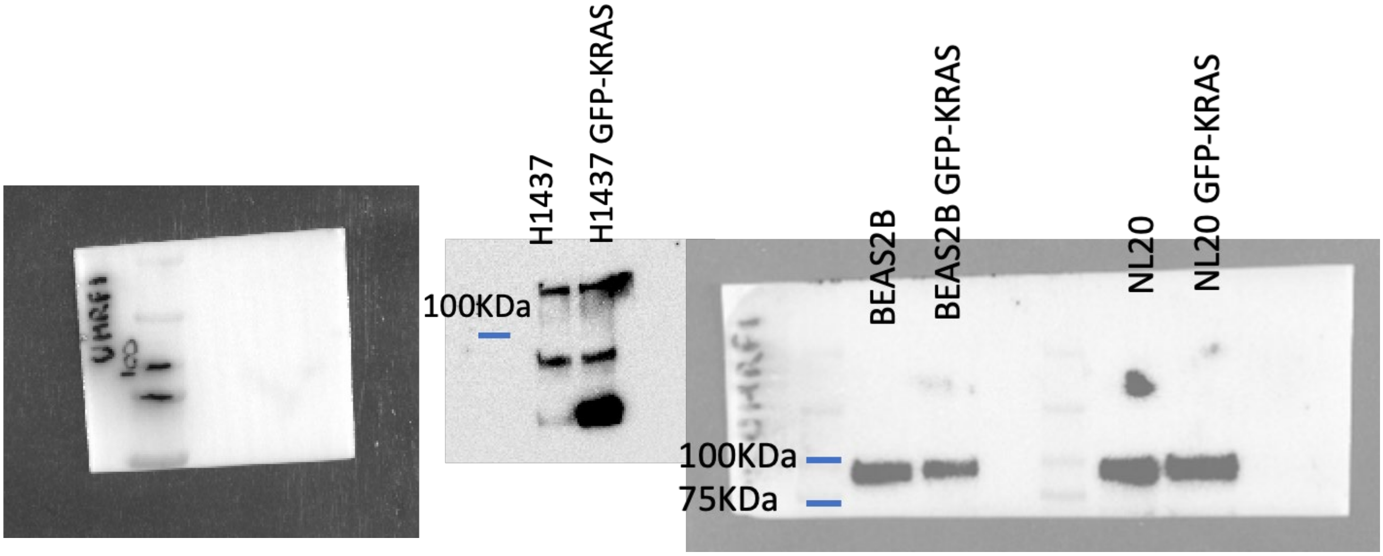

GFP (66002-1-Ig) 1:2,000

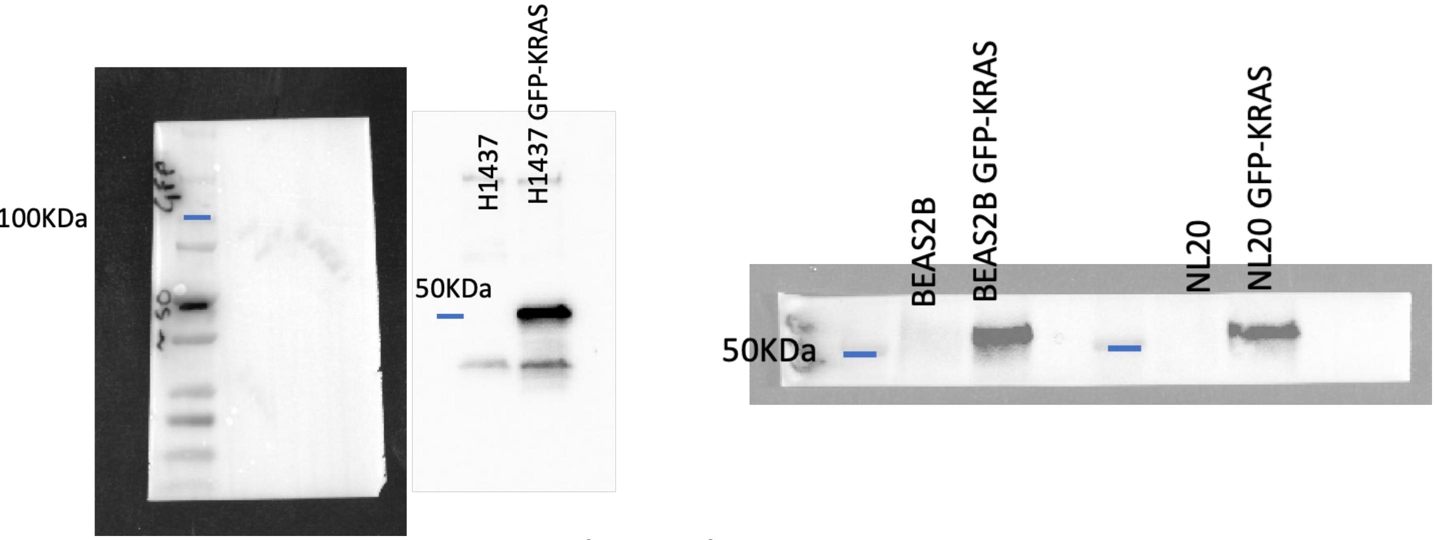

GAPDH (ab9485) 1:5000

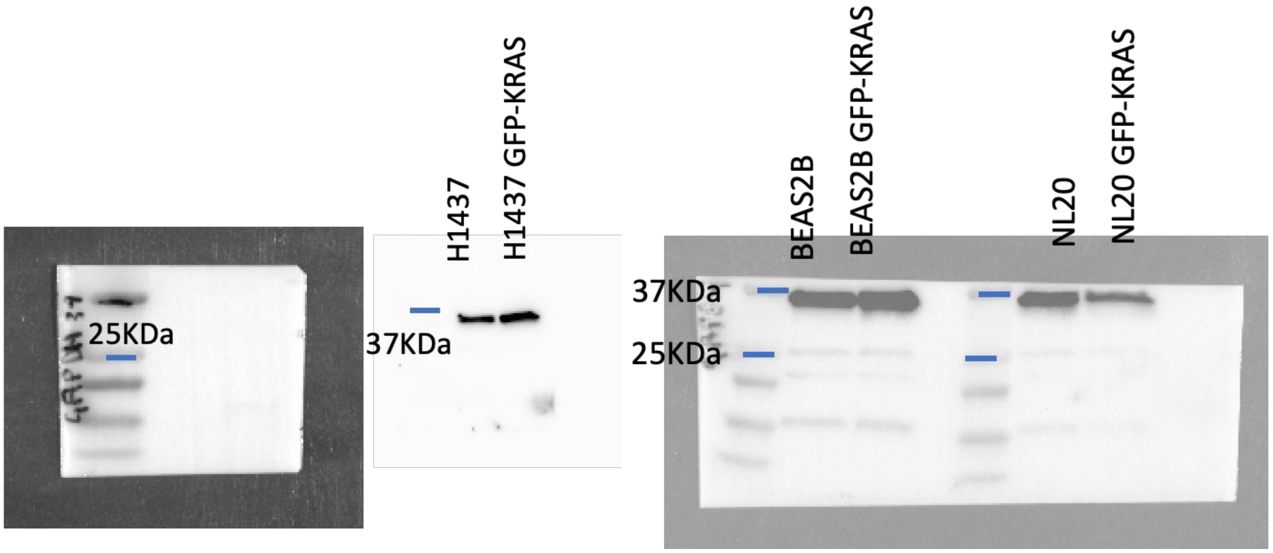

Supplement: Supplementary file 1 — Supplementary Information [file 41467_2023_39591_MOESM1_ESM.pdf]
