# Supplementary material for: UHRF1 is a mediator of KRAS driven oncogenesis in lung adenocarcinoma
Source: Nat Commun. 2023 Jul 5;14:3966. doi: 10.1038/s41467-023-39591-2 (PMC10322837; doi:10.1038/s41467-023-39591-2)
Supplement: Supplementary file 3 — Description of Additional Supplementary Files [file 41467_2023_39591_MOESM3_ESM.pdf]

Description of Additional Supplementary Files

File Name: Supplementary Data 1
Description: List of targets included in the "KRAS effectors" shRNA library.

File Name: Supplementary Data 2
Description: List of targets included in the "KRAS interactors" shRNA library.

File Name: Supplementary Data 3
Description: Sequences of shRNAs used in primary spheroid screens.

File Name: Supplementary Data 4
Description: Supplementary Data 4: Results of RNAi screens in primary spheroids (3D) or adherent cells (2D). Results represented as Log2 fold change between endpoint (T2) and initial timepoint (T1).

File Name: Supplementary Data 5
Description: Pathways significantly enriched in UHRF1-depleted cells (siUHRF1) compared to control cells (siNeg). GSEA analysis performed on differentially methylated regions using gene sets from GO terms, KEGG and Reactome.

File Name: Supplementary Data 6
Description: Differential gene expression analysis between UHRF1-depleted cells (siUHRF1) and control cells (siNeg).

File Name: Supplementary Data 7
Description: Differential gene expression analysis between KRAS-depleted cells (siKRAS) and control cells (siNeg).

File Name: Supplementary Data 8
Description: TSGs significantly hypomethylated in UHRF1-depleted cells (siUHRF1) compared to control cells (siNeg).

File Name: Supplementary Data 9
Description: CRISPR library for the minipool TSG screen.

File Name: Supplementary Data 10
Description: Results of the minipool CRISPR screen in A549 cells.

File Name: Supplementary Data 11
Description: TSGs significantly correlated with UHRF1 expression in LUAD samples (TCGA).

File Name: Supplementary Data 12
Description: List of publicly available datesets used in the study.
