# Supplementary material for: UHRF1 is a mediator of KRAS driven oncogenesis in lung adenocarcinoma
Source: Nat Commun. 2023 Jul 5;14:3966. doi: 10.1038/s41467-023-39591-2 (PMC10322837; doi:10.1038/s41467-023-39591-2)
Supplement: Supplementary file 5 — Reporting Summary [file 41467_2023_39591_MOESM5_ESM.pdf]

nature portfolio

Corresponding author(s): Kaja Kostyrko
Alejandro Sweet-Cordero

Last updated by author(s): May 26, 2023

Reporting Summary

Nature Portfolio wishes to improve the reproducibility of the work that we publish. This form provides structure for consistency and transparency in reporting. For further information on Nature Portfolio policies, see our Editorial Policies and the Editorial Policy Checklist.

Statistics

For all statistical analyses, confirm that the following items are present in the figure legend, table legend, main text, or Methods section.

n/a	Confirmed	
☐	☒	The exact sample size (*n*) for each experimental group/condition, given as a discrete number and unit of measurement
☐	☒	A statement on whether measurements were taken from distinct samples or whether the same sample was measured repeatedly
☐	☒	The statistical test(s) used AND whether they are one- or two-sided<br>*Only common tests should be described solely by name; describe more complex techniques in the Methods section.*
☐	☒	A description of all covariates tested
☐	☒	A description of any assumptions or corrections, such as tests of normality and adjustment for multiple comparisons
☐	☒	A full description of the statistical parameters including central tendency (e.g. means) or other basic estimates (e.g. regression coefficient) AND variation (e.g. standard deviation) or associated estimates of uncertainty (e.g. confidence intervals)
☐	☒	For null hypothesis testing, the test statistic (e.g. *F*, *t*, *r*) with confidence intervals, effect sizes, degrees of freedom and *P* value noted<br>*Give P values as exact values whenever suitable.*
☒	☐	For Bayesian analysis, information on the choice of priors and Markov chain Monte Carlo settings
☒	☐	For hierarchical and complex designs, identification of the appropriate level for tests and full reporting of outcomes
☐	☒	Estimates of effect sizes (e.g. Cohen's *d*, Pearson's *r*), indicating how they were calculated

Our web collection on statistics for biologists contains articles on many of the points above.

Software and code

Policy information about availability of computer code

Data collection	Cell proliferation data was collected using Incucyte S3 software (v2019a, Essen BioScience). Images were taken on DMi8 fluorescence microscope (Leica). Flow cytometry data was collected using Accuri C6 (BD Biosciences) and analyzed using FlowJo (v10.7.1) . Real-time PCR was performed using C1000 Touch Thermal Cycler (BioRad). No software was used for multi-omics data collection.
Data analysis	Scripts, codes, and selected publicly available datasets for multi-omic analysis, which includes the import of raw sequences, preprocessing, filtering, statistical analysis, and final reports, are accessible in a public repository: https://github.com/ahdee/Kostyrko_2023. All analysis was completed with R (3.6.2), where indicted). Minimal R packages includes, edgeR (3.28.1 or 3.28.1), Minfi (1.32), EpiDISH (2.2.2), limma package (3.42.2), missMethyl (1.20.4), DMRcate (2.0.7), survminer (0.4.6.999), survival (3.1.11), DGCA (1.0.2) and methylGSA (1.4.9). Cytoscape (3.8.2) and clueGO (2.5.9) were also used for pathway analysis.

For manuscripts utilizing custom algorithms or software that are central to the research but not yet described in published literature, software must be made available to editors and reviewers. We strongly encourage code deposition in a community repository (e.g. GitHub). See the Nature Portfolio guidelines for submitting code & software for further information.

Data

Policy information about availability of data

All manuscripts must include a data availability statement. This statement should provide the following information, where applicable:

- Accession codes, unique identifiers, or web links for publicly available datasets
- A description of any restrictions on data availability
- For clinical datasets or third party data, please ensure that the statement adheres to our policy

All raw sequence data has been deposited in GEO as a SuperSeries under the accession number GSE198450 (https://www.ncbi.nlm.nih.gov/geo/query/acc.cgi?acc=GSE198450). This SuperSeries is composed of the following SubSeries:
GSE198289 UHRF1 is a mediator of KRAS driven oncogenesis in lung adenocarcinoma [RNA-seq]
GSE198446 UHRF1 is a mediator of KRAS driven oncogenesis in lung adenocarcinoma [epic_methyl]
GSE209923 UHRF1 is a mediator of KRAS driven oncogenesis in lung adenocarcinoma [shRNA]
GSE233401 UHRF1 is a mediator of KRAS driven oncogenesis in lung adenocarcinoma [CRISPR_screen]

Human research participants

Policy information about studies involving human research participants and Sex and Gender in Research.

Reporting on sex and gender	N/A
Population characteristics	N/A
Recruitment	N/A
Ethics oversight	N/A

Note that full information on the approval of the study protocol must also be provided in the manuscript.

Field-specific reporting

Please select the one below that is the best fit for your research. If you are not sure, read the appropriate sections before making your selection.

☒ Life sciences ☐ Behavioural & social sciences ☐ Ecological, evolutionary & environmental sciences

For a reference copy of the document with all sections, see nature.com/documents/nr-reporting-summary-flat.pdf

Life sciences study design

All studies must disclose on these points even when the disclosure is negative.

Sample size	The sample size was determined based on our previous experience and power calculations as described in Zheng et al., 2013 (PMID: 23845442). For in vivo experiments sample sizes were determined based on Jackson et al., 2005 (PMID: 16288016).
Data exclusions	No data was excluded
Replication	Each in vivo experiment presented in the paper was repeated in multiple mice (n > 5 per arm). Technical replicates for in vitro experiments were done with n=3. At least 3 independent experiments were performed for in vitro assays with consistent results.
Randomization	For all in vivo experiments mice were randomly allocated to experimental groups. Animals were allocated to groups to ensure similar age and sex distribution between groups. For in vitro experiments wells in cell culture dishes were allocated to different treatment groups at random.
Blinding	There were no therapeutic treatment studies where blinding would be appropriate.

Reporting for specific materials, systems and methods

We require information from authors about some types of materials, experimental systems and methods used in many studies. Here, indicate whether each material, system or method listed is relevant to your study. If you are not sure if a list item applies to your research, read the appropriate section before selecting a response.

Materials & experimental systems

n/a	Involved in the study
☐	☒ Antibodies
☐	☒ Eukaryotic cell lines
☒	☐ Palaeontology and archaeology
☐	☒ Animals and other organisms
☒	☐ Clinical data
☒	☐ Dual use research of concern

Methods

n/a	Involved in the study
☒	☐ ChIP-seq
☐	☒ Flow cytometry
☒	☐ MRI-based neuroimaging

Antibodies

Antibodies used

UHRF1 (sc-373750, Santa Cruz Biotechnology), KRAS (Op24, Millipore), Cyclin D1 (ab134175, Abcam), actin (Sigma, A5316), Cyclin B1 (ab32053, Abcam), GAPDH (no. 9485, Abcam), Ki67 (ab15580, Abcam), biotinylated secondary horse anti-mouse IgG biotinylated antibody (Vector Laboratories, BA-2000), goat anti-rabbit AF488 secondary antibody (ThermoFisher Scientific, A-11008), goat anti-mouse AF647 secondary antibody (ThermoFisher Scientific, A-21235), pH2AX (Cell Signaling, #9718), p-ERK (Cell Signaling, #4370), Erk1/2 (Cell Signaling, #4695), p-AKT (#13038, Cell Signaling), AKT (#75692, Cell Signaling), AF647 goat anti-rabbit (Thermo Fisher Scientific, #A21245).

Validation

UHRF1, KRAS and Cyclin D1 antibodies were validated using western blot on cells treated with UHRF1, KRAS and CyclinD1 siRNAs, respectively (Fig. 5h, Extended Data Fig. 3). Ki67 and pH2AX antibodies have been validated for immunofluorescence, relevant citations can be found on the manufacturer's websites: https://www.abcam.com/products/primary-antibodies/ki67-antibody-ab15580.html and https://www.cellsignal.com/products/primary-antibodies/phospho-histone-h2a-x-ser139-20e3-rabbit-mab/9718. Cyclin B1, pERK1/2, ERK1/2, pAKT, AKT, actin and GAPDH antibodies have been validated for western blotting, relevant citations can be found on manufacturer's websites: https://www.abcam.com/products/primary-antibodies/cyclin-b1-antibody-y106-ab32053.html, https://www.cellsignal.com/products/primary-antibodies/phospho-p44-42-mapk-erk1-2-thr202-tyr204-d13-14-4e-xp-rabbit-mab/4370, https://www.cellsignal.com/products/primary-antibodies/p44-42-mapk-erk1-2-137f5-rabbit-mab/4695, https://www.cellsignal.com/products/primary-antibodies/phospho-akt-thr308-d25e6-xp-rabbit-mab/13038, https://www.cellsignal.com/products/primary-antibodies/akt1-d9r8k-rabbit-mab/75692?site-search-type=Products&N=4294956287&Ntt=%2375692&fromPage=plp&_requestid=1030454, https://www.sigmaaldrich.com/US/en/product/sigma/a5316, https://www.abcam.com/products/primary-antibodies/gapdh-antibody-loading-control-ab9485.html.

Eukaryotic cell lines

Policy information about cell lines and Sex and Gender in Research

Cell line source(s)

Human NSCLC cell lines (NCI-H1437 #CRL-5872, NCI-H1568 #CRL-5876, NCI-H1650 #CRL-5883, NCI-H1975 #CRL-5908, NCI-H460 #HTB-177, NCI-H1792 #CRL-5895, NCI-H2009 #CRL-5911, NCI-H23 #CRL-5800, NCI-H358 #CRL-5807, A549 #CCL-185), HBEC cell lines (NL20 #CRL-2503, BEAS-2B #CRL-9609), and HEK 293T #CRL-3216 cells were obtained from the ATCC. LKR10 cells were derived from the KRAS G12D mouse model (DuPage et al, 2011). The cells were a gift from Julien Sage (Stanford School of Medicine, Stanford, CA, USA).

Authentication

Commercial human cell lines were positively authenticated using CellCheck 9 - human (9 Marker STR Profile and Inter-species Contamination Test) from Idexx Bioanalytics (https://www.idexxbioanalytics.com). Mouse cell lines used in the study were not authenticated.

Mycoplasma contamination

All cell lines were tested for mycoplasma contamination by PCR (IDEXX BioResearch). All cell lines were negative for Mycoplasma sp.

Commonly misidentified lines
(See ICLAC register)

No commonly misidentified cell lines were used in this study.

Animals and other research organisms

Policy information about studies involving animals; ARRIVE guidelines recommended for reporting animal research, and Sex and Gender in Research

Laboratory animals

8-10 week old NSG mice were used for xenograft experiments. 5-10 week old Uhrf1fl/fl KrasLSL-G12D Trp53fl/fl (UKP), Uhrf1fl/fl KrasLSL-G12D (UK), KrasLSL-G12D Trp53fl/fl (KP), and KrasLSL-G12D (K) mice were used for genetically engineered mouse experiments. The mice were housed in the HDFCCC animal facility at UCSF in individually ventilated microisolator cages with automatic watering system purified using reverse osmosis. The cages are on a 12/12 hour light/dark cycle. All feed is pre-irradiated. Temperature is maintained between 68-72 degrees and humidity is maintained at 30-70%.

Wild animals

This study did not involve wild animals

Reporting on sex

Sex was not considered in this study. Both male and female animals were used in animal experiments.

Field-collected samples

This study did not involve field-collected samples

Ethics oversight	All the procedures involving mice were are approved by the Institutional Animal Care and Use Committee (IACUC) at UCSF (protocol #AN15761).

Note that full information on the approval of the study protocol must also be provided in the manuscript.

Flow Cytometry

Plots

Confirm that:

☒ The axis labels state the marker and fluorochrome used (e.g. CD4-FITC).

☒ The axis scales are clearly visible. Include numbers along axes only for bottom left plot of group (a 'group' is an analysis of identical markers).

☒ All plots are contour plots with outliers or pseudocolor plots.

☒ A numerical value for number of cells or percentage (with statistics) is provided.

Methodology

Sample preparation	For flow cytometry experiments cells were seeded into 6-well plates. After 3-5 days medium was collected and stored on ice. Cells were trypsinized and transferred to collected medium. Number of total cells was counted and 1x10^6 cells were transferred to new tubes. For cell cycle analysis the cells were washed in cold PBS, fixed by adding ice-cold 70% ethanol drop-wise with gentle vortexing and fixed for 30min at -20°C. After fixation, cells were washed in cold PBS, then incubated in the dark for 30 min at 37°C in 500ul of staining solution containing PBS, 200µg/ml RNAse A (Qiagen, 17,500 U, #19101) and 40µg/ml propidium iodide (Invitrogen, #P3566). After incubation the cells were centrifuged, resuspended in 500ul of PBS and analyzed by flow cytometry on Accuri C6 (BD Biosciences). For apoptosis analysis the cells were stained with APC Annexin V Apoptosis Detection Kit with propidium iodide (BioLegend #640932) following manufacturer's instructions and analyzed by flow cytometry on Accuri C6 (BD Biosciences).
Instrument	Flow cytometry data was acquired on Accuri C6 (BD Biosciences)
Software	Flow cytometry data was analyzed using FlowJo (v10).
Cell population abundance	Cell sorting was not performed in this study.
Gating strategy	FSC and SSC were used to gate out single cell populations. Negative and positive staining populations were determined based on single-stained controls.

☒ Tick this box to confirm that a figure exemplifying the gating strategy is provided in the Supplementary Information.
